# The MerR-like protein BldC binds DNA direct repeats as cooperative multimers to regulate *Streptomyces* development

**DOI:** 10.1038/s41467-018-03576-3

**Published:** 2018-03-19

**Authors:** Maria A. Schumacher, Chris D. den Hengst, Matthew J. Bush, T. B. K. Le, Ngat T. Tran, Govind Chandra, Wenjie Zeng, Brady Travis, Richard G. Brennan, Mark J. Buttner

**Affiliations:** 1 Department of Biochemistry, Duke University School of Medicine, Durham, NC 27710 USA; 2 Department of Molecular Microbiology, John Innes Centre, Norwich Research Park, Norwich, NR4 7UH UK

## Abstract

Streptomycetes are notable for their complex life cycle and production of most clinically important antibiotics. A key factor that controls entry into development and the onset of antibiotic production is the 68-residue protein, BldC. BldC is a putative DNA-binding protein related to MerR regulators, but lacks coiled-coil dimerization and effector-binding domains characteristic of classical MerR proteins. Hence, the molecular function of the protein has been unclear. Here we show that BldC is indeed a DNA-binding protein and controls a regulon that includes other key developmental regulators. Intriguingly, BldC DNA-binding sites vary significantly in length. Our BldC-DNA structures explain this DNA-binding capability by revealing that BldC utilizes a DNA-binding mode distinct from MerR and other known regulators, involving asymmetric head-to-tail oligomerization on DNA direct repeats that results in dramatic DNA distortion. Notably, BldC-like proteins radiate throughout eubacteria, establishing BldC as the founding member of a new structural family of regulators.

## Abstract (web summary)

BldC regulates the onset of differentiation in Streptomycetes by a yet unknown molecular mechanism. Using a combination of structural, biochemical and in vivo approaches, the authors show that BldC controls the transcription of several developmental regulators and unravel its DNA binding mode.

## Introduction

S*treptomyces* are ubiquitous, primarily soil-dwelling filamentous bacteria that undergo a complex developmental transition from vegetative growth to the production of reproductive aerial hyphae, which differentiate into chains of exospores^1–5^. Entry into development coincides with the biosynthesis of numerous secondary metabolites that serve as our most abundant source of clinically important antibiotics and provide other medically important drugs such as anticancer agents and immunosuppressants^6–8^. As a consequence, there is considerable interest in understanding the mechanisms that control this developmental transition. Genetic studies identified the regulatory loci that control entry into development, which are called *bld* (bald) genes because null mutations in these loci prevent the formation of fuzzy aerial hyphae for one of two diametrically opposite reasons: either because they block differentiation (mutations in activators) or because they cause precocious hyper-sporulation, bypassing the formation of aerial hyphae (mutations in repressors). The three Bld regulators that fall into this latter class are BldD, BldO, and BldC^4,9–12^. BldD is a transcriptional repressor that sits at the top of the developmental hierarchy and is regulated by cyclic-di-GMP (c-di-GMP). c-di-GMP mediates the dimerization of two BldD protomers, leading to DNA binding^4,9,13–15^. In this way, c-di-GMP drives repression of the large BldD regulon of sporulation genes, extending vegetative growth and inhibiting the hypha-to-spore transition^4,9,13,15^. Unlike the pleiotropic regulator BldD, BldO prevents entry into development by acting as the dedicated repressor of a single key developmental target, *whiB*^10,11^. Like BldD and BldO, BldC acts as a repressor to sustain vegetative growth, and so deletion of *bldC* causes the premature onset of sporulation^12^. To date, however, there has been no mechanistic insight into the way BldC prevents entry into development.

*bldC* encodes a 68-residue protein that is predicted to contain a winged helix-turn-helix (wHTH) motif, suggesting it may be involved in DNA binding. This wHTH shows the strongest sequence similarity to those of the MerR family of transcriptional activators^16^. The basic structure of classical MerR proteins is a dimer consisting of two identical subunits, each composed of an N-terminal wHTH DNA-binding domain, a C-terminal effector-recognition domain and an interconnecting linker region that consists of a long α helix that interacts with the same helix in the other subunit, forming an antiparallel coiled-coil responsible for homodimerization^17–24^. MerR proteins share significant sequence similarity only within their DNA-binding domains; as different family members bind different effectors, their C-terminal domains are variable and show little, if any, similarity to one another. Promoters controlled by classical MerR family regulators have unusually long (19–20 base pairs (bp) vs. 17 bp) spacers between their −10 and −35 promoter elements such that these motifs are misaligned for binding by the σ factor of the RNA polymerase (RNAP) holoenzyme, rendering the promoters inactive for transcription. Structures for several canonical MerR proteins have been solved in their apo and DNA-bound activated forms and reveal a conserved mode of transcription activation. Specifically, classical MerR proteins bind operators in the spacer between the −10 and −35 regions and when the C-terminal effector-recognition domain binds its cognate ligand, the transcription factor untwists and shortens the DNA, realigning the −10 and −35 sequences to allow RNAP holoenzyme to bind and activate transcription^17–24^.

While BldC appears to contain a MerR-like wHTH, it is to date, the only known MerR-like protein that consists entirely of a wHTH with no obvious effector or oligomerization domain. Interestingly, bioinformatic analysis shows that there are numerous BldC homologs in both Gram-negative and Gram-positive bacteria, suggesting that BldC represents a large family of putative DNA-binding proteins^16^. Despite this, the roles of BldC and its homologs remain unknown. Using a battery of structural, biochemical, and in vivo approaches, we show here that BldC functions as a pleotropic regulator of *Streptomyces* development by employing a unique mode of DNA binding for a transcription factor that involves asymmetric head-to-tail oligomerization on DNA direct repeats of varying number with concomitant distortion of the DNA. This mode of DNA binding defines a new family of transcription regulatory proteins that we designate the BldC family.

## Results

### Identification of BldC binding sites in vivo

**Fig. 1:**
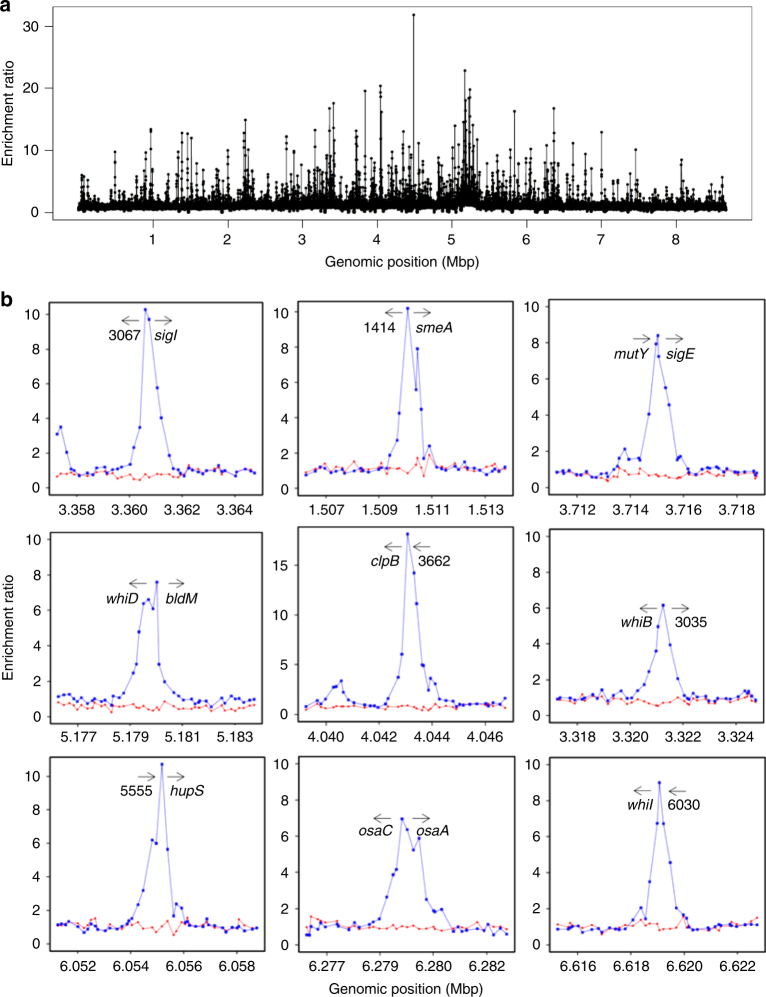
The *S. coelicolor* BldC regulon. **a** Chromosome-wide distribution of BldC binding sites identified by ChIP-chip analysis. DNA obtained from immunoprecipitation of BldC was labeled with Cy3 and hybridized to DNA microarrays together with a total DNA control labeled with Cy5. Data are plotted as Cy3/Cy5 ratios (*y*-axis), as a function of chromosome location (*x*-axis). **b** ChIP-chip data for nine selected BldC targets in WT *S. coelicolor* and the *S. coelicolor ΔbldC* mutant (blue and red dots, respectively). Plots span approximately 8 kb of DNA sequence. Gene names or identifiers (SCO numbers) are indicated below the arrows, which indicate gene orientation

The presence of a putative wHTH in BldC suggests it functions in DNA binding and transcription regulation. Therefore, to identify possible BldC binding sites in vivo, we performed chromatin immunoprecipitation-microarray (ChIP-chip) using a polyclonal BldC antibody^16^. Figure 1a,b and Supplementary Data 1 present the results from two independent biological experiments. A congenic *bldC* null mutant was used as a control to eliminate any potential signals that might arise from cross-reaction of the antibody with other transcription factors. ~280 BldC-specific peaks were detected (*P*-value <= 0.05, *P*-value based on empirical Bayes moderated *t*-statistic test), scattered across the genome (Fig. 1a and Supplementary Data 1). 25 BldC target genes encode regulatory proteins themselves (Supplementary Table 1), implying a pleiotropic role for BldC in *Streptomyces* development. Indeed, promoter sites bound by BldC are found upstream of genes encoding many key transcriptional regulators of the *Streptomyces* developmental cascade including *bldM*, *whiB*, *whiD*, *whiH*, *whiI*, *sigF*, and *bldC* itself, in addition to others encoding proteins involved in chromosome segregation and condensation during sporulation such as *smeA*-*sffA*^25^ and *hupS*^26^ (Fig. 1b and Supplementary Data 1) and genes that influence antibiotic production, specifically the conservon *cvnA1* (ref. ^27^) and the serine-threonine protein kinase, *afsK*^28^. In parallel work, we used qRT-PCR to examine the expression of 5 key BldC targets (*whiI*, *smeA*, *whiD*, *sigF*, and *hupS*) in *Streptomyces venezuelae* comparing the wild type (WT) and a congenic *bldC* mutant. In each case, the gene was expressed earlier in the *bldC* mutant than in the WT, indicating that the transcription of these genes is directly regulated by BldC^12^. Both BldC and BldD inhibit entry into development^4,9,12^, and previously we identified ~160 genes controlled by BldD using ChIP-chip^4,9,15^. Comparison of genes bound by BldC or BldD showed only a small overlap of 15 genes/operons bound by both proteins (Supplementary Table 2). However, these include the key developmental genes *whiB*, *whiD*, *bldM*, *smeA-sffA*, *bldC*, and *cvnA1*.

### BldC binding to the *whiI* promoter

**Fig. 2:**
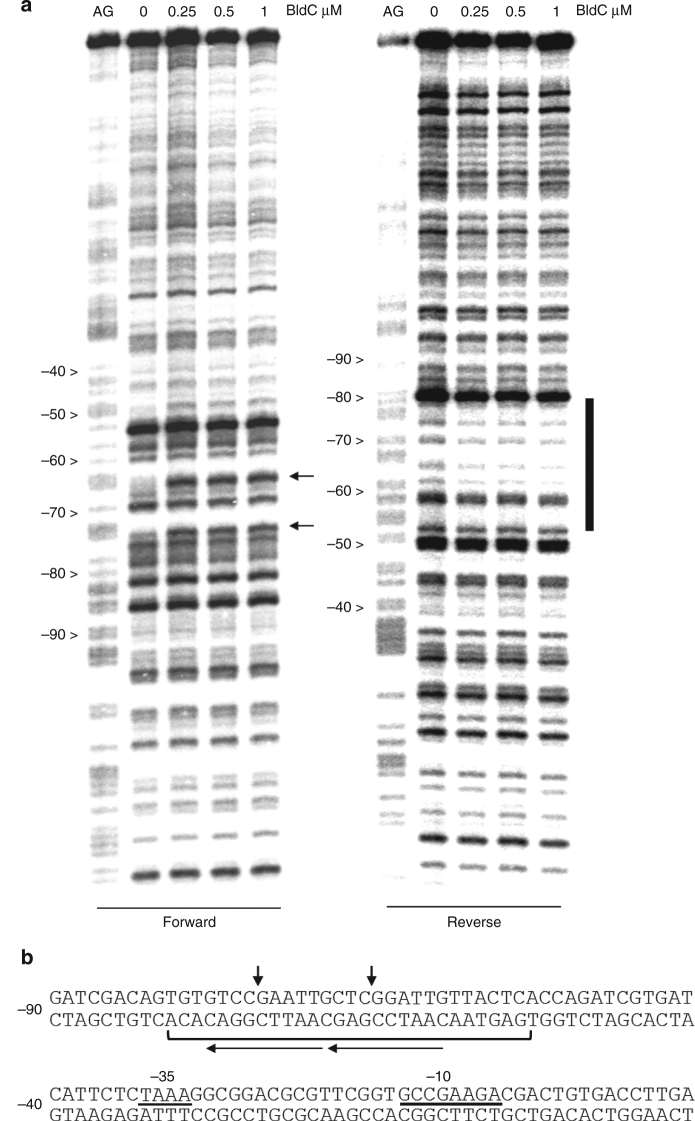
BldC binding to the *whiI* promoter region. **a** DNase I footprinting analysis. 5′ end-labeled probes were incubated with increasing amounts of BldC (indicated in µM above the lanes) and subjected to DNase I footprinting analysis as described in Methods. Footprints are flanked on the left-hand side by Maxam and Gilbert sequence ladders (AG). Horizontal black arrows indicate sites of enhanced DNase I cleavage on the forward strand and the vertical bar indicates the region of DNase I protection on the reverse strand. **b** Summary of DNase I footprinting results presented in **a**. The bracket indicates the protected region and the vertical black arrows indicate sites of enhanced DNase I cleavage. The numbers indicate the distance to the 5′ end of the *whiI* transcript. The direct repeats shown to be bound by BldC in a head-tail orientation are indicated by arrows (with arrows pointing in the head-tail direction)

To glean insight into the DNA binding mode of BldC, we performed DNase I protection studies on the promoter regions of two important BldC-regulated loci: *whiI* and the *smeA-ssfA* operon. *whiI* is among the key developmental targets of BldC (Fig. 1b and Supplementary Data 1), being essential for the late stages of sporulation^4,29,30^. *whiI* encodes an orphan response regulator that activates its regulon by forming a functional heterodimer with a second orphan response regulator, BldM^4,30^. DNase I protection studies on the *whiI* promoter show that BldC protects an ~30 bp region from –50 to –80, an unusual location from which to exert repression. Nevertheless, RNA analysis shows that *whiI* transcription comes on much earlier in a *bldC* mutant than in the WT^12^ indicating it is regulated by BldC. The BldC-protected region harbors an imperfect direct repeat and two sites of enhanced DNase I cleavage are observed on the top strand, suggesting that BldC binding may distort DNA (Fig. 2). Notably, the enhanced cleavages also appear periodic in nature, falling on approximately the same face of the DNA and within the imperfect direct repeats (Fig. 2). To gain more insight into the specific sequence and minimal length requirement for high affinity DNA binding by BldC, we performed EMSA analyses employing a variety of DNA duplexes based on the *whiI* site that ranged from 16 to 30 bp (Supplementary Fig. 1). These analyses identified the 22-bp sequence, top strand 5′-TGTGTCCGAATTGCTCGGATTG-3′ as the shortest DNA duplex that bound BldC. Binding to this sequence by BldC was subsequently optimized by introducing 3 base changes, which also allowed the DNA duplex to be shortened to 20 bp, 20R sym, with a top strand sequence, 5′-TGTCCGAATTGTCCGAATTG-3′ (Supplementary Fig. 1). Two of these changes are naturally present in the *whiI* promoter of the alternative model species *S. venezuelae* and the amino acid sequences of BldC from *Streptomyces coelicolor* and *S. venezuelae* are identical.

### BldC binding to the *smeA-sffA* promoter

**Fig. 3:**
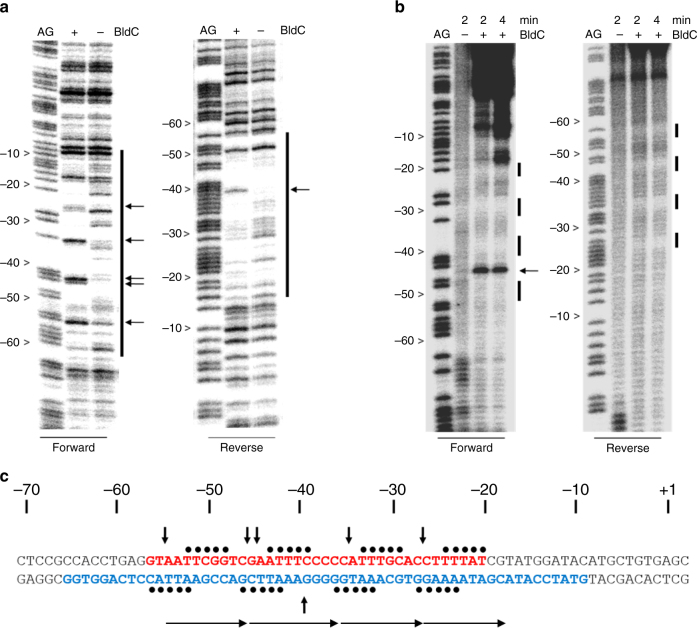
BldC binding to the *smeA-ssfA* promoter region. **a** DNase I and **b** hydroxyl radical footprinting analysis. 5′ end-labeled probes were incubated in the presence (1 µM) or absence of BldC and subjected to footprinting analysis as described in Methods. Footprints are flanked on the left-hand side by Maxam and Gilbert sequence ladders (AG). Horizontal black arrows indicate sites of enhanced cleavage and vertical bars indicate regions of DNase I protection. **c** Summary of the footprinting results presented in **a** and **b**. The filled black circles indicate the repeat pattern of bases protected from hydroxyl radical attack. The bases colored red and blue indicate the DNase I-protected sequences on the forward and reverse strands, respectively, and the vertical black arrows indicate the repeat pattern of DNase I hypersensitive sites seen on the top strand in the presence of BldC. The numbers indicate the distance to the 5′ end of the *smeA* transcript. Direct repeat motifs are indicated by head-to-tail arrows

Another key target regulated by BldC is the *smeA-sffA* operon (Fig. 1b and Supplementary Data 1). The *smeA-sffA* operon encodes a DNA translocase (SffA) involved in chromosome segregation during sporulation that is specifically targeted to septa by the small membrane protein SmeA^25^. Deletion of *smeA-sffA* results in a defect in spore chromosome segregation and has pleiotropic effects on spore maturation^25^. Strikingly, in contrast to the ~30 bp BldC-protected region in the *whiI* promoter, BldC protects a much larger region of the *smeA-sffA* promoter, extending approximately from –60 to –10 relative to the transcription start site. A regular pattern of hypersensitive sites on the top strand is observed in the footprint (Fig. 3a,b). While the BldC-binding site in the *whiI* promoter contains two direct repeat elements, the BldC binding site within the *smeA-sffA* promoter reveals four similar repeats, but arranged in the opposite orientation, 5′ to 3′ (see Fig. 3a,b). To explore BldC binding to the *smeA-sffA* promoter further, a finer analysis was undertaken using hydroxyl radical footprinting. BldC protects four regularly spaced 5-bp tracts, with one helical turn of the DNA between one tract and the next (Fig. 3b,c), suggesting that four molecules of BldC bind the *smeA-ssfA* promoter. However, only one shift is observed in EMSA analyses of BldC binding to the *smeA-sffA* site, suggesting that BldC binds this extended site cooperatively (Supplementary Fig. 2).

### BldC-DNA structure reveals MerR-like structural family

**Fig. 4:**
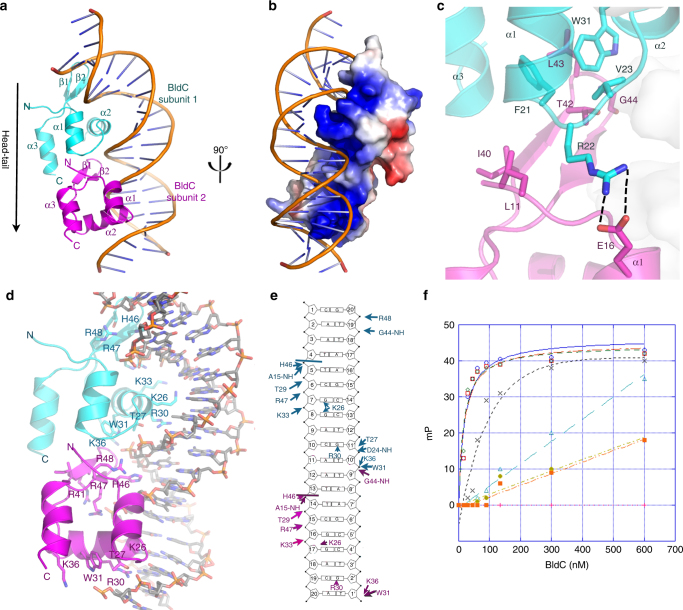
Structure of the BldC-*whiI* opt DNA complex. **a** Overall BldC-*whiI* opt structure with one BldC subunit colored cyan and the other magenta. The secondary structural elements are labeled. The head-tail directionality of BldC binding is indicated by an arrow. **b** Electrostatic surface representation of the structure rotated by 90° relative to **a**. Blue and red represent electropositive and electronegative regions, respectively. **c** Contacts important in BldC head-tail dimerization on the DNA. The side chains that mediate dimer formation are shown as sticks. The contacts between Arg22 and Glu16 from apposing subunits are shown as dashed lines. **d** BldC-DNA contacts. Residues that contact the DNA are shown as sticks and labeled. **e** Schematic ladder diagram showing the contacts between BldC and DNA. Note, for clarity, the 2 bp overhangs that are not contacted by BldC are not included. **f** FP binding isotherms of various BldC proteins binding to a fluorescently labeled *whiI* opt 22mer. Experiments were preformed in technical triplicate and the error between measurements noted. Shown are representative binding isotherms for WT His_6_-BldC (red squares), His_6_-Semet BldC(L43M-L58M) (green diamonds), Tag-free WT BldC (blue circles), His_6_-BldC(E16R) (black crosses), His_6_-BldC(G44E) (orange squares), His_6_-BldC(H46A) (light blue triangles) and His_6_-BldC(R30A) (yellow diamonds). The resulting *K*_d_s for these curves are 18.2 nM ± 4.8 nM, 19.6 nM ± 4.8 nM, 16.5 nM ± 4.0 nM, 140 nM ± 63 nM, no saturable binding (NB), NB and NB, respectively. The *y*-axis and *x*-axis are mP and BldC concentration (nM), respectively

The mechanism by which BldC binds sites with variable numbers of direct repeat elements is unclear, but it is likely to differ from that used by other MerR transcriptional regulators, which function as symmetric dimers that bind palindromic DNA sites. Consistent with this hypothesis, the BldC protein does not contain a canonical MerR family dimerization domain. Further, unlike classical MerR proteins, size exclusion chromatography (SEC) studies showed that apo BldC is a monomer (Supplementary Fig. 3). Thus, to deduce the molecular mechanism underlying DNA binding and recognition by BldC, we determined the structure of *S. coelicolor* BldC bound to a 22 bp double-stranded DNA based on the optimized *whiI* binding site (*whiI* opt). The structure was solved by single wavelength anomalous diffraction (SAD) using data collected from a selenomethionine-substituted BldC(L43M-L58M)-*whiI* opt DNA crystal (WT BldC-*whiI* opt produced the same crystals) and refined to *R*_work_/*R*_free_ values of 22.3%/26.8% to 3.28 Å resolution (Supplementary Table 3 and Supplementary Fig. 4). Notably, the structure reveals that BldC forms a head to tail (head-tail) dimer on the DNA whereby each BldC protomer binds to a 9 bp direct repeat with the recognition helix of the HTH motif interacting in the major groove and the wing in the minor groove (Fig. 4a,b). Because the BldC dimer is asymmetric it binds head-tail with a specific directionality, which is 5′ to 3′ with the sequence, 5′-CAATTCGGACAATTCGGACA-3′ (Fig. 4d,e and Supplementary Fig. 1). Formation of the head-tail BldC dimer buries ~600 Å^2^ of protein surface from solvent. The oligomer interface is largely hydrophobic with residues Phe21, Val23, and Trp31 packing against the side chains of Leu11′, Ile 40′, Thr42′, Leu43′, and Gly44′ (where ′ indicates the other subunit of the DNA-stabilized dimer) (Fig. 4c and Supplementary Fig. 5). Hydrogen bonds/electrostatic contacts between the side chains of Arg22 and Glu16′ help fasten the two subunits together (Fig. 4c and Supplementary Fig. 5). While the buried surface area of the DNA-bound BldC dimer is significant for such a small protein it is much less than that observed in stable dimers, likely explaining why BldC must be anchored to its cognate DNA to assemble the asymmetric dimer.

The DNA binding mode exhibited by BldC is notably distinct from that used by classical MerR proteins, all of which bind palindromic DNA sites as symmetric dimers^17–25^ (Supplementary Fig. 6a). The head-tail binding of the two BldC protomers bends the *whiI* site by ~50°. The helical twist (35.1°) and rise per base pair (3.25 Å) of the BldC-bound DNA remains similar to B-DNA (36.0° and 3.32 Å, respectively). However, the minor and major groove widths are significantly distorted: the wing-bound, AT-rich minor groove has an average width of 9.3 Å (compared to 12.0 Å for B-DNA) whereas the minor groove outside this region is widened significantly (14.5 Å). The major groove in which the recognition helix docks is expanded to 19.4 Å compared 17.2 Å for B-DNA. Despite employing a DNA binding mode different from classical MerR proteins, the BldC wHTH indeed harbors a fold that is highly similar to these proteins. The BldC structure follows the typical MerR topology: α1 (BldC residues 14–21), α2 (25–34), β1 (38–42), β2 (45–48), and α3 (51–60) (Fig. 4a). Moreover, DALI searches indicate that the BldC wHTH shows significant structural similarity to the wHTH domains of the MerR proteins with which it superimposes with root mean square deviations (rmsds) of 1.7–2.2 Å for 45–58 corresponding Cα atoms. The superimposition of BldC onto the wHTH of the MerR protein MtaN underscores the homology between the wHTHs but also the lack of the dimerization coiled-coil in BldC (Supplementary Fig. 6b).

### Molecular basis of DNA binding specificity of BldC

Each BldC subunit makes a large number of phosphate and base contacts, allowing for high affinity BldC binding. The side chains of BldC residues Thr27, Thr29, and Lys33 and the amide nitrogens of Ala15 and Asp24 from the HTH anchor the wHTH on the DNA via phosphate contacts to both DNA strands. The Trp31 side chain also interacts with the phosphate backbone via its Nε and induces a kink in the DNA (Fig. 4d,e). The BldC wing is particularly rich in basic residues with Arg47 and Arg48 providing key contacts that aid in docking the wing into the minor groove. The combined DNA-binding region of the two interacting BldC subunits also presents a striking electropositive surface that complements the bound DNA (Fig. 4b).

The DNA-binding specificity of BldC is mediated by residues from both its wing and its HTH. Residues from the wing recognize the AT-rich narrowed minor groove. The His46 side chain fits within the narrowed groove and makes numerous hydrogen bonds and van der Waals contacts with sugar and phosphate moieties (Fig. 4d). The importance of the AT-rich motif in BldC binding sites was assessed by fluorescence polarization (FP) binding assays. These studies showed that replacement of the AATT motif with GGCC abrogated DNA binding (Supplementary Fig. 7). Major groove base contacts are provided by BldC residues Arg30 and Lys26, both located on the recognition helix of the HTH. The relatively low resolution of the structure prevents a highly detailed description of protein-DNA contacts and the possible role of waters in the protein-DNA interface. However, despite the low resolution, the electron density for the Arg30 side chain is clear and reveals that it provides highly specific, bidentate hydrogen bonds to Gua2′ and Gua11′ (Fig. 4d,e and Supplementary Fig. 8). Simultaneously, the Arg30 guanidinium groups stack with the bases of Thy1′ and Thy10′, which are located 5′ of the contacted guanine (Fig. 4d,e and Supplementary Fig. 9). This type of specific protein-DNA contact has been called a 5′-pyrimidine-guanine-3′ (5′-YpG-3′) interaction and it arises from the inherent flexibility of pyrimidine-guanine steps. A survey of the protein database by Glover and coworkers showed that diverse classes of DNA-binding motifs utilize this DNA recognition element^31–33^. This type of contact mediates specific binding to two bases whereby an arginine side chains interacts with the major groove face of the 5′-guanine nucleobase and also contacts the unstacked preceding pyrimidine^31,32^. The side chain of BldC residue Lys26 also makes base contacts. However, the electron density for the Lys26 side chain is less well resolved than that of the Arg30 side chain suggesting it is conformationally flexible. Moreover, while the Lys26 side chain is within hydrogen bonding distance of major groove guanines from bps 7, 8, and 17 (Fig. 4e), it could also interact with thymine O4 atoms in other sequences, suggesting that it may not dictate complete readout specificity of major groove bases. Indeed, binding studies revealed that replacement of the guanines at bp8 and bp17 with thymine (Fig. 4e) had little effect on binding (app *K*_d_ of WT = 20 nM compared to 50 nM ± 1.1 nM for the T mutant), while replacement of the guanines with cytosines resulted in a nearly 6-fold reduction in binding (app *K*_d_ = 115 nM ± 10 nM) (Supplementary Fig. 7). Hence, the flexible contacts provided by this residue may contribute to the range of sequences that BldC can bind within select promoters.

Thus, the analysis of the BldC-*whiI* opt structure indicates that there are two main DNA elements specifically recognized by BldC; an AT-rich region and a C-G, 4 base pairs downstream from the AT-rich region, which is recognized by Arg30. The consensus direct repeat for binding by each BldC protomer is, therefore, AATTXXXX(C), where the C represents the C-G bp containing the guanine recognized by Arg30. Because the BldC dimer binds direct repeats in a head-tail manner, its interaction with the DNA is also directional. As a result, BldC binds *whiI* opt and the WT *whiI* promoter in a reverse orientation, i.e., the BldC dimer is arranged head-tail, 5′ to 3′ on the bottom strand of the DNA (Figs. 2, 4a).

### Probing the BldC-DNA structural model

The BldC-DNA structure revealed several unexpected findings for a MerR-like protein, in particular a direct repeat DNA binding mode involving the formation of a head-tail dimer. Thus, to further test the structural model, we mutated several residues observed to be important for DNA binding in the structure as well as those involved in dimer formation and carried out FP DNA binding assays. The WT protein, with or without the his_6_-tag, and the selenomethionine BldC(L43M-L58M) protein bound the 22 bp *whiI* opt DNA with essentially the same *K*_d_ (~20 nM) (Fig. 4f). An E16R mutation, which would disrupt the dimer stabilizing contact with Arg22 (Fig. 4c), resulted in a ~10-fold reduction in DNA binding while a G44E substitution, which places a large, negatively, charged residue at the hydrophobic interface of the dimer and hence is predicted to disrupt the head-tail dimer, led to complete loss of DNA binding (Fig. 4f). Individual substitution of residues His46 and Arg30, which make key contacts to the minor and major grooves, respectively, with a glutamate (H46E) or an alanine (R30A), also essentially abrogated DNA binding (Fig. 4f). Thus, these combined data support the unusual mode of DNA binding observed in the BldC-DNA structure. Importantly, circular dichroism (CD) analyses showed that all mutant BldC proteins were properly folded and exhibited the same CD spectrum as WT BldC (Supplementary Fig. 10).

### BldC-*smeA-ssfA* structure shows extended protein-DNA filament

**Fig. 5:**
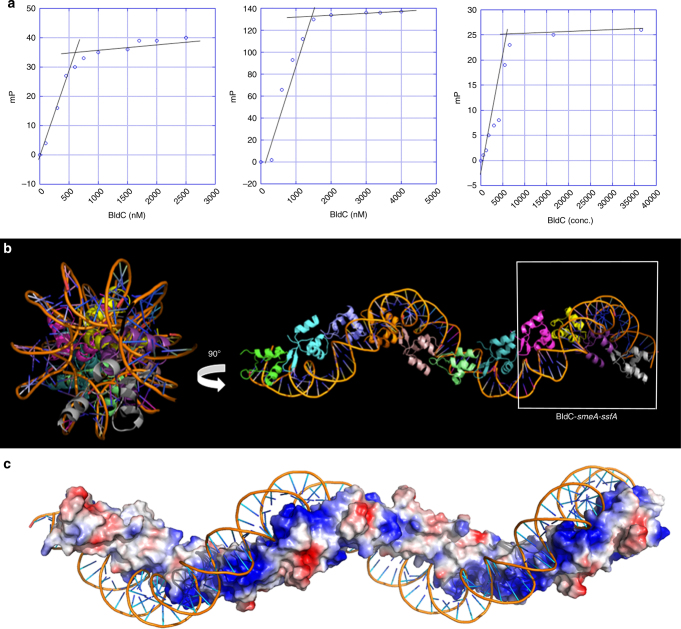
BldC binds DNA to form extended protein-nucleic acid superstructural filaments. **a** FP-based stoichiometry binding experiments performed with fluoresceinated 22mer opt, 36mer opt (containing 4 binding repeats), and the *smeA-ssfA* BldC site, each experiment was conducted in technical triplicate with a representative analysis shown for each. For the experiments, DNA was present at a concentration 10-fold above the *K*_d_s, which are 20, 20, and 60 nM, respectively. Binding was observed as increasing mPs until saturation, at which the mPs no longer increase. The inflection point of each binding isotherm represents the subunit protein concentration at saturation. This occurred at BldC: DNA duplex stoichiometries of 2:1, 4:1, and 4:1, respectively. **b** Structure of the BldC-*smeA-ssfA* promoter complex. The DNA packs pseudo-continuously. The unit corresponding to the BldC-*smeA-ssfA* complex is boxed. Two views are shown. Left is a view looking down the axis of the structure and right shows the view rotated 90°. The structure shows how BldC could bind promoters with longer sites than present in the *smeA-ssfA* promoter. **c** Electrostatic representation of the structure with blue and red, representing electropositive and electronegative regions. Notably, the continuous protein superstructure also forms a continuous electropositive stripe that tracks along the DNA

The head-tail mode of DNA binding observed in the BldC-*whiI* opt structure (Fig. 4) could permit the formation of continuous protein–DNA complexes through extension at one or both ends of the asymmetric dimer, which could explain the mechanism which BldC utilizes to interact with longer DNA binding sites such as that of the *smeA-sffA* promoter. The BldC protected region in the *smeA-ssfA* promoter region contains four AATTXXXX(C) motifs similar those in the *whiI* site (5′-AATTCGGTC-3′, 5′-GATTTCCCC-3′, 5′-CATTTGCAC-3′, and 5′-CTTTTATCG-3′, where the C-G bp recognized by Arg30 is underlined) (Fig. 3). But, in the *smeA-ssfA* promoter these repeats proceed in a 5′ to 3′ direction and the last repeat does not conform to the consensus (Fig. 2). However, conservation of the exact consensus sequence does not appear to be critical for specific binding, in particular for the AT-rich region, as it is the narrowing of the minor groove caused by the AT-rich nature of that sequence that is important. This so-called indirect readout^33^ based on the shape of the minor groove as well as the pliability in contacts afforded by the Lys36 side chain in the major groove likely allows BldC to bind DNA sites that harbor differences from the consensus. As a result, BldC might be able to interact with sites that contain repeats that diverge from the consensus in longer DNA sites provided there are consensus sites to enable the docking of subunits that can then enable binding by other protomers. This binding mode predicts that multiple BldC subunits might bind a DNA site such as the *smeA-ssfA* operator. To address this issue, we determined the *K*_d_ and stoichiometry of BldC binding to the 22mer *whiI* opt site, to an extended 36-mer opt site that contains four perfect direct repeats and to the *smeA-ssfA* site. BldC bound these DNA sites with apparent affinities (*K*_d_s) of 20, 20, and 60 nM, respectively, and displayed binding stoichiometries (BldC subunits per DNA duplex) of 2:1, 4:1, and 4:1, respectively (Fig. 5a). These data, therefore, support the hypothesis that multiple BldC subunits can bind cooperatively to extended DNA sites.

**Fig. 6:**
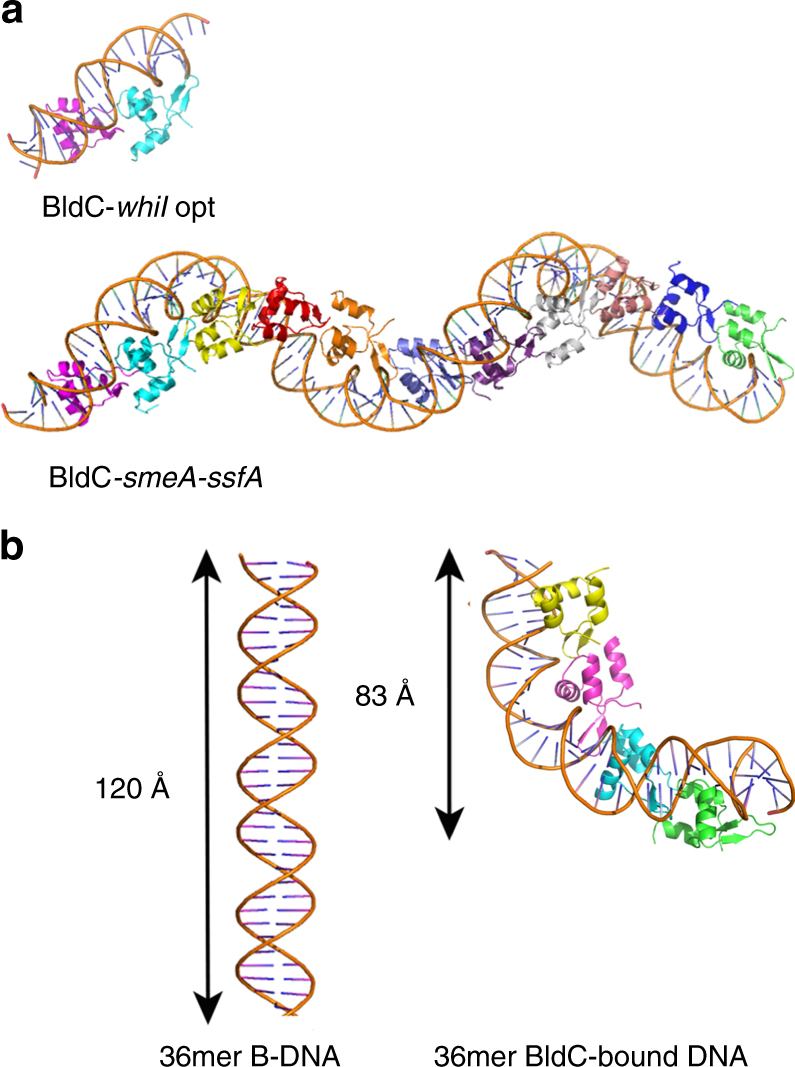
Comparison of B-DNA to BldC bound DNA. **a** Comparison of the BldC-*whiI* opt and BldC-*smeA-ssfA* structures. BldC forms identical head-tail dimers in both structures. **b** The cooperative binding of multiple BldC subunits significantly distorts and shortens the DNA

To deduce the molecular basis for this extended binding we next determined the structure of BldC in complex with an 18mer *smeA-ssfA* DNA fragment that contained the first two repeats of the *smeA-ssfA* BldC binding site, 5′-GCAATTCGGTCGAATTTC-3′ (Fig. 3b). The DNA was constructed to generate pseudo-continuous packing of the DNA, which was indeed generated in the crystals (see Methods). The structure was solved by molecular replacement (MR) and refined to *R*_work_/*R*_free_ values of 20.9%/27.8% to 3.09 Å resolution (Supplementary Table 3). The structure reveals a striking extended protein-nucleic acid filament with multiple BldC subunits arranged in the same head-tail manner as observed in the BldC-*whiI* opt structure (Figs. 5b,c and 6a). The asymmetric BldC dimer contacts and DNA docking mode in this structure are identical to those observed in the BldC-*whiI* opt structure. Because the DNA is pseudocontinuous, the register of the DNA sequence in this structure was unclear. Indeed, due to the continuous stacking of the DNA, it was possible that the 9 bp sites bound by each BldC subunit are statistically disordered (half of the time containing one 9 bp sequence and the other half, the other 9 bp sequence). That is the stacking of the 18mer, end to end, creates two BldC repeats with consensus repeats, AATTCGGTC and GAATTTCGC, which conserves the CG bp recognized by Arg30 (underlined). To address the possibility that the DNA may be statistically disordered, X-ray intensity data were collected on a crystal grown with DNA containing a thymine to 5-iododeoxyuracil substitution (5′-GTAAXTCGGTCGAATTTC-3′, where X is 5-iododeoxyuracil). An electron density difference map revealed no strong electron density peaks for the iodine indicating that the DNA is statistically disordered.

While the DNA contacts in this structure are statistically disordered, the structure reveals how multiple BldC subunits can bind a promoter site with multiple cognate repeats to create an extended protein superstructure. This superstructure results in a continuous electropositive patch that tracks along the length of the DNA (Fig. 5c). This regular superstructure would also explain the periodic hypersensitivity revealed in the DNase I protection experiments. The continuous coating of its DNA site by BldC binding also suggests one obvious mechanism of transcription repression via sterically restricting access of RNAP to the promoter. This mechanism would be at play despite the directionality of the BldC head-tail oligomer along the DNA. The binding of multiple BldC subunits along the DNA also leads to severe DNA distortion and shortening: the BldC subunits reduce the length of the DNA by a third when compared to canonical B-DNA (Fig. 6b). Consequently, the DNA has an overall positive writhe.

## Discussion

Our combined data reveal that while BldC harbors a MerR-like wHTH motif, it binds DNA in a manner distinct from classical MerR family proteins^17–24^. Interestingly, recent structural studies on other non-MerR proteins have revealed wHTH domains with structural similarity to the MerR wHTH. For example, structural analyses of the master regulators of nitrogen metabolism in *B. subtilis*, TnrA and GlnR, revealed that both proteins have a MerR-like wHTH motif, however both TnrA and GlnR lack the coiled-coil domain of classical MerR proteins but instead contain N-terminal extensions and unstructured C-terminal regions^34^. Instead of using the canonical coiled-coil, TnrA and GlnR dimerize upon DNA binding through residues in their N-terminal regions and use their C-terminal regions to interact with glutamine synthetase (GS)^34^. In common with canonical MerR proteins, however, both TnrA and GlnR bind as homodimers to palindromic DNA sites. Structural homology searches also uncovered architectural DNA binding proteins that do not function in transcription, including RacA and Xis, which harbor wHTH structures similar to MerR proteins^35–37^.

Interestingly, BldC appears similar to classical MerR proteins in that it makes few base specific contacts. Indeed, the structures of BmrR, MtaN, and CueR bound to DNA revealed that only one to two residues make base contacts per subunit^17,19,20^. This is consistent with MerR proteins employing indirect readout as part of their DNA binding mechanisms. Notably, unlike canonical MerR regulators, the MerR-like protein RacA was observed to make YpG contacts using an arginine that is equivalent to the BldC residue Arg30^36^. However, aside from this similarity, RacA and BldC utilize very different DNA binding mechanisms. In particular, the interaction of RacA with its GC-rich DNA site does not lead to global DNA bending but causes a significant widening of its entire minor groove. By contrast, BldC binding induces significant global bending in its bound DNA and a narrowing of the AT-rich minor grooves. Also, unlike RacA, which binds DNA as a symmetric dimer, BldC binds direct repeats in a head-tail manner. To our knowledge, BldC is the only MerR-like transcription regulator that binds direct repeats and employs only its wHTH domain for DNA contact and oligomerization. Bacterial transcription regulators that bind DNA direct repeats appear less common than those that bind palindromic DNA sites as symmetric dimers. Well characterized examples of bacterial regulators, other than BldC, that bind direct repeats include some members of the large family of two component response regulators (RR)^38–40^. RRs harbor N-terminal receiver domains flexibly attached to DNA-binding domains. More than 95% of characterized RRs utilize a HTH DNA-binding domain while the remaining 5% of RRs employ a LytTR domain for DNA binding. Structures have been obtained for both these types of DNA-binding domain, revealing that some RRs, such as PhoP, PmrA, and AgrA,  bind DNA direct repeats. Studies on the RR AcrA revealed that, like BldC, it can bind multiple tandem repeats^41^, however a structure is not yet available of the full length AcrA bound to cognate DNA elements.

Finally, bioinformatic analyses reveal that small, BldC-like proteins radiate throughout the domain of bacteria, being present in ~500 species, including actinomycete pathogens (*Mycobacterium tuberculosis* and *Corynebacterium diphtheriae*), commercially important actinomycetes (*Corynebacterium glutamicum*), and other Gram-positive (e.g*., Bacillus thuringiensis*) and Gram-negative bacteria (e.g., *Sinorhizobium meliloti* and *Porphyromonas gingivalis*) (Supplementary Fig. 11a). Sequence analysis shows strong conservation of key DNA-binding and head-tail oligomerization residues in BldC-like proteins in the actinomycetes. More distant members of the BldC family also contain hydrophobic residues in regions involved in head-tail dimer formation (Supplementary Fig. 11a). However, key residues involved in BldC head-tail dimer formation are not conserved in classical MerR family proteins. In particular, Leu43, which interacts with Trp31ʹ and Phe21ʹ to form the key hydrophobic core of the BldC head-tail dimer, is replaced by a charged residue (glutamic or aspartic acid) in ZntR, CueR, MtaN, and BmrR, or by a polar residue (serine, tyrosine or histidine) in SoxR, MerR, and TipAL (Supplementary Fig. 11b). Modeling indicates that such charged or polar residues would prohibit formation of a head-tail dimer as they would be placed proximal to hydrophobic side chains. These findings suggest that, unlike classical MerR proteins, the other BldC-like proteins identified bioinformatically are likely to have the same DNA binding mode observed for BldC itself, and that they can likewise form extended protein-nucleic acid superstructures. In that regard, it is interesting to note that some BldC-like orthologues in the database are annotated as possible resolvases/integrases, consistent with our finding that BldC shares strong structural similarity to Xis. These findings suggest formerly overlooked, possible evolutionary connections between MerR transcription regulators and integrases and DNA architectural proteins.

## Methods

### Cell culture, strains, plasmids, and oligonucleotides

Strains and plasmids used in this study are listed in Supplementary Table 4. Oligonucleotides used are listed in Supplementary Table 4 unless given in the text. *Streptomyces coelicolor* strains were grown on mannitol soy flour (MS, also known as SFM) or R5 solid media^42^. Strains used for ChIP-chip analysis were grown in a 1:1 mixture of yeast extract-malt extract (YEME) and tryptic soy-broth (TSB) liquid media^42^ at 30 °C.

### Chromatin immunoprecipitation-microarray studies

To carry out the ChIP-chip experiments, *S. coelicolor* M600 was grown in duplicate for 15 h in YEME/TSB liquid medium and a single culture of the congenic *bldC* null mutant strain J2166 was grown in the same way as the control. Formaldehyde was added to cultures at a final concentration of 1% (v/v) and incubation was continued for 30 min. Glycine was then added to a final concentration of 125 mM to stop the cross-linking. Cultures were left at room temperature (RT) for 5 min before the mycelium was harvested and washed twice in PBS buffer pH 7.4. Each mycelial pellet was resuspended in 0.5 ml lysis buffer (10 mM Tris HCl pH 8.0, 50 mM NaCl) containing 15 mg/ml lysozyme and protease inhibitor (Roche Applied Science) and incubated at 25 °C for 1 h. Subsequently, 0.5 ml IP buffer (100 mM Tris HCl pH 8, 250 mM NaCl, 0.5% Triton-X-100, 0.1% SDS) containing protease inhibitor was added and samples were chilled on ice. Samples were sonicated for 7 cycles of 15 s each at 10 microns to shear the chromosomal DNA into fragments ranging from 300–1000 bp in size. Samples were centrifuged twice at 34,000 × *g* at 4 °C for 15 min to clear the cell extract, after which 10 µl of cell extract was set aside for total DNA extraction. The remainder (900 µl) was incubated with 45 µl protein A-sepharose (Sigma) for 1 h on a rotating wheel to clear from non-specifically binding proteins. Samples were then centrifuged for 15 min at 4 °C and 34,000 × *g* to remove the beads. Supernatants were incubated with 75 μl crude anti-BldC antiserum (76 mg/ml; custom made [16]) overnight at 4 °C with rotation. Subsequently, 90 µl protein A-sepharose was added to precipitate BldC and incubation was continued for 4 h. Samples were centrifuged for 5 min and the pellets were washed twice with 0.5x IP buffer, then twice with 1x IP buffer. Each pellet was incubated overnight at 65 °C in 150 µl IP elution buffer (50 mM Tris HCl pH 7.6, 10 mM EDTA, 1% SDS) to reverse cross-links, and 10 µl of the total cell extract control was treated in the same way. Samples were centrifuged at 34,000 × g for 5 min to remove the beads. Each pellet was re-extracted with 50 µl TE buffer (10 mM Tris HCl pH 7.4, 1 mM EDTA) and the resulting supernatant was combined with the primary supernatant and incubated with 0.2 mg/ml Proteinase K (Roche) for 2 h at 55 °C. The resulting samples were extracted with phenol-chloroform and further purified using QiaQuick columns (Qiagen). DNA was eluted in 50 µl EB buffer and quantified using a NanoDrop spectrophotometer (Thermo Scientific).

DNA labeling and hybridization to DNA microarrays were carried out by using a Bio-Prime kit (Invitrogen). Briefly, for these experiments 800 ng of the total and immuno-precipitated DNA was labeled with Cy5-dCTP and Cy3-dCTP, respectively. Using an Agilent Technologies hybridization oven, the labeled DNA was hybridized to high-density DNA microarrays representing the genome of *S. coelicolor*, which were designed and manufactured by Oxford Gene Technology (OGT). Following washing, the arrays were read using an Agilent Technologies scanner and the Cy5 and Cy3 signals were quantified using Agilent’s Feature Extraction software.

From the data files received from OGT, columns relevant for further analysis were extracted using a bespoke Perl script. This resulted in three files (two for the WT replicates and one for the control) containing the following columns: name of the probe; start of the probe on the *S. coelicolor* genome; end of the probe on the *S. coelicolor* genome; median green (Cy3) signal intensity; median red (Cy5) signal intensity; the probe sequence. Each of the three files obtained above was read into R and the Cy3 and the Cy5 signals were transformed to their log2 values and normalized using the normalizeQuantiles function of the Bioconductor package limma. Log ratios of the Cy3–Cy5 signals were then calculated and normalized to the average signal intensity using the loess function of R. The three sets of normalized log ratios obtained were put in a single data frame in R. After making the design and contrasts matrices appropriate for the data, the limma functions lmFit, contrasts.fit and eBayes were applied in sequence. Finally, the function toptable was used to generate a table of probes listing enrichment ratios of the WT relative to the *bldC* mutant control, ordered by *P*-values (determined by empirical Bayes moderated *t*-statistic test) adjusted using the method of Benjamini and Hochberg^43^. Signals around probes with *P*-values less than or equal to 0.05 were then plotted and inspected manually to determine whether they were part of a signal peak or not.

### Purification of BldC

Plasmid pIJ6838, a pET15b (Novagen) derivative expressing his-tagged full-length BldC, was introduced into *E. coli* BL21(DE3) and grown in Luria-Bertani liquid medium. Following IPTG induction for 3 h at 30 °C in the presence of 0.3 mM IPTG, a microfluidizer was used to disrupt cells. The lysate (in Buffer A: 25 mM Tris HCl pH 7.5, 300 mM NaCl, 5% glycerol) was loaded onto a nickel-nitrilotriacetic acid (Ni-NTA) column. The his-tagged BldC was eluted using increasing concentrations of imidazole in Buffer A. For protein used for crystallization trials, an additional SEC purification step was included. The protein was >95% pure after this step.

### In vitro DNA binding assays

DNase I footprinting experiments were carried out similar to previous studies^44^ and according to the method supplied with the Sure Track footprinting kit (GE Healthcare). Specifically, DNA fragments were prepared by PCR using the oligonucleotides pairs 6029_F1 and 6029_R1 and 1415_F2 and 1415_R2 to generate DNA probes spanning the BldC-binding sites in the *whiI* and *smeA-ssfA* promoters, respectively. Oligonucleotides were first end-labeled with T4 polynucleotide kinase (GE Healthcare) and [γ-^32^P]-ATP as described by the manufacturer. Binding reactions were performed in a total volume of 40 μl containing 10 mM Tris HCl pH 7.8, 150 mM NaCl, 2 mM dithiothreitol, 1 µg poly(dI-dC) (Roche), and 10% (v/v) glycerol in the presence of approximately 110,000 cpm of the DNA probe. Following DNase I treatment and purification, products were separated on denaturing 6% (w/v) polyacrylamide gels and visualized using a FLA-7000 phosphorimager (Fujifilm).

Hydroxyl radical footprinting was performed using a protocol similar to that described previously^45^. Specifically, the binding reactions and probes were identical to those used for the DNase I footprinting experiments, but in a total volume of 25 μl and in the absence of glycerol. After 20 min at RT, Fe(NH_4_)_2_(SO_4_)_2_, and EDTA were added to final concentrations of 10 µM and 20 µM, respectively, followed by the addition of sodium ascorbate to 1 µM and H_2_O_2_ to 0.015% (v/v). Reactions were incubated for a further 1–4 min at RT before quenching the reaction with 9 μl stop solution (27.77 mM thiourea, 27.77 mM EDTA, 1 M Na acetate, containing 25 μg yeast tRNA). Samples were precipitated with three volumes of ethanol, re-dissolved in stop solution, phenol–chloroform extracted and analyzed in the same way as the DNase I footprints.

Electrophoretic mobility shift assays (EMSAs) were carried out using a DNA probe containing the BldC-binding site from the *smeA-sffA* promoter, radiolabelled on the forward strand. The probe was generated by PCR using the oligonucleotides 1415_F2 and 1415_R2. For EMSA experiments, increasing concentrations of BldC protein was added from 0.004 μM to 1.2 μM. The reaction samples were incubated for 20 min at RT and then run on 5% polyacrylamide gels.

### Crystallization and determination of BldC-DNA structures

The his-tag was removed from BldC prior to crystallization trials using a thrombin capture cleavage kit (Qiagen). Crystals of BldC bound to an optimized version of the *whiI* DNA site were obtained by hanging drop vapor diffusion. The DNA (termed *whiI* opt) contained TT and AA overhangs (top strand: 5′-TTCAATTCGGACAATTCGGACA-3′). For crystallization, BldC was concentrated to 0.5 mM before mixing with 0.5 mM double stranded *whiI* opt DNA and this complex then combined 1:1 with a crystallization solution consisting of 30% MPD, 0.1 M sodium acetate pH 5.0, and 25% PEG 1500, and placed over a 1 ml well containing the crystallization solution. Crystals were obtained at RT and took several weeks to grow to maximum size. To obtain phase information, a double mutant, BldC(L43M-L58M) was constructed and used to produce selenomethionine protein and isomorphous crystals grown with *whiI* opt DNA. The crystals diffracted to 5 Å, however, the diffraction limit was extended by dragging a crystal to the edge of the drop with a loop and allowing it to dehydrate before placing it in the cryo-stream. This permitted data collection beyond 3.5 Å resolution. The crystals take the hexagonal space group, P6_1_22. SAD X-ray intensity data were collected for a selenomethionine BldC(L43M-58M)-*whiI* opt crystal to 3.28 Å resolution at the selenium peak at the advanced light source (ALS) beamline 8.3.1. The data were processed in MOSFLM and scaled using SCALA. Autosol was used to determine heavy atom positions and perform density modification^46^. Due to the high solvent content (65%) density modification produced an experimental map of excellent quality, which was used to manually build the model. There are two BldC subunits and one DNA duplex in the crystallographic asymmetric unit (ASU). Once constructed, the model was subjected to multiple rounds of refinement using Phenix^46^, rebuilding using O^47^ and validation with MolProbity^48^, resulting in final *R*_work_/*R*_free_ values of 22.3%/26.8% to 3.28 Å resolution (Supplementary Table 3). The analysis of the DNA structure was carried out using w3DNA^49^.

The BldC-*whiI* opt structure revealed that each BldC subunit binds a 9 bp DNA site arranged as direct repeats. Hence, to obtain a BldC-*smeA-ssfA* promoter DNA complex, WT BldC was mixed with a DNA site that contained the first two BldC binding sites from the *smeA-ssfA* promoter anticipating that pseudocontinuous DNA packing would generate the *smeA-ssfA* site. Crystals of the complex were obtained by mixing 0.5 mM BldC (subunit) with 0.5 mM *smeA-ssfA* DNA and concentrating the mixture 3-fold using a 30 kDa microcon concentrator. The concentrated mixture was crystallized by mixing it 1:1 with 35% PEG 400, 0.1 M CaCl_2_, 0.1 M Hepes 7.5. Crystals were produced at RT and grew to maximum size within a week. The crystals take the tetragonal space group, P4_1_22. X-ray intensity data were collected at ALS beamline 8.3.1 and processed with MOSLM and scaled with SCALA. The structure was solved by molecular replacement (MR) using the previously determined P6_1_22 structure with the DNA truncated to 18 bp as a search model^50^. As the structure contained multiple copies of BldC and DNA, the structure was solved in a stepwise procedure. First, an initial solution consisting of two BldC subunits bound to a 18mer DNA site was obtained using MolRep. This solution was then used as a static model to find an additional 2 BldC-18mer complex. This strategy was followed until 10 BldC subunits and five 18mers were included in the model. This model was subjected to rigid body refinement followed by positional refinement in Phenix^46^. After this initial refinement, the *R*_work_ was 39% and the *F*_o_−*F*_c_ difference map revealed electron density for an additional BldC subunit and 9 bp of DNA, which were then added to the model. Thus, the asymmetric unit contains 11 BldC promoters bound to eleven 9 bp sites. Because the DNA was pseudocontinuous, the register of the DNA sequence was unclear. Indeed, because of the continuous stacking of the DNA, it was possible that the 9 bp sites bound by each BldC subunit are statistically disordered (half the time containing one 9 bp and the other half, the other 9 bp sequence). To address this possibility, X-ray intensity data were collected on a crystal grown with DNA containing a thymine to 5-iododeoxyuracil substitution (5′-GTAAXTCGGTCGAATTTC-3′, where X = 5-iododeoxyuridine). A difference map (*F*_wt_−*F*_iodo_) revealed no clear peaks indicating that the DNA is statistically disordered. For simplicity, each 9 bp sequence was constructed as 5′-AATTCGGAC-3′. After multiple cycles of refinement, rebuilding in O and validation in MolProbity^48^, the *R*_work_/*R*_free_ values converged to 20.9%/27.8%, respectively. Data collection and refinement statistics are listed in Supplementary Table 3.

### Fluorescence polarization-based DNA binding experiments

Fluorescence polarization (FP) experiments were performed using a PanVera Beacon 2000 FP system at 25 °C. 5′-Fluroesceinated oligonucleotides were used for DNA binding experiments with WT BldC and BldC mutants. For each FP experiment, WT BldC or BldC mutant protein were titrated into 0.995 mL of reaction buffer (25 mM Tris, pH 7.5, 150 mM NaCl) containing 1 nM fluoresceinated oligonucleotide. Fluoresceinated oligonucleotides used in the experiments include the *whiI* opt (top strand: 5′-F-TTCAATTCGGACAATTCGGACA-3′ where 5′-F denotes the 5′ fluorescein label), 36mer optimized site (top strand: 5′-F-ATTCGGACAATTCGGACAATTCGGACAATTCGGACA-3′) and the *smeA-ssfA* promoter site (top strand: 5′-F-TAATTCGGTCGAATTTCCCCCATTTGCACCTTTTATCGTA-3′). The sequences of the three mutant *whiI* opt sites used for experiments shown in Supplementary Fig. 7 are: 5′-F-TTCGGCCCGGACGGCCCGGACA-3′ (top strand), 5′-F-TTCAATTCGCACAATTCGCACA-3′ (top strand), and 5′-F-TTCAATTCGTACAATTCGTACA-3′ top strand). The binding studies on BldC ( ±  the his-tag), selenomethionine BldC(L43M-L58M), and BldC mutants with the F-*whiI* opt DNA showed that the his-tag had no effect on DNA binding nor did the L43M-L58M mutations and selenomethionine substitution. FP experiments examining binding of the 36mer and *smeA-ssfA* sites were conducted with WT BldC and all data were fit using Kaleigraph.

### Stoichiometry determination experiments

To determine the binding stoichiometry of BldC to the *whiI* opt, 36mer opt, and *smeA-ssfA* site, the buffer and conditions were identical to those used in the FP binding affinity determination experiments except that the concentration of the DNA site was increased to 10-fold above the *K*_d_ (by using a solution containing 1 nM F-DNA and an amount of unlabeled DNA necessary to achieve a concentration 10-fold above *K*_d_) thereby ensuring stoichiometric binding. For each oligonucleotide, BldC was titrated into the binding solution and the graph of the resulting data shows a linear increase in the observed mP until saturation with DNA, after which the mP values showed no further increase. The inflection point reflects the binding stoichiometry for each DNA site (obtained from the concentration values).

### Size exclusion chromatography

SEC was used to probe the molecular weight of BldC using a using a HiLoad 26/600 Superdex 75 prep grade column. Experiments were performed in a buffer containing 200 mM NaCl, 5% glycerol, 20 mM Tris HCl pH 7.5 and 1 mM β-mercaptoethanol (BME). For SEC analysis BldC was included in the buffer at a concentration of 10 μM.

### CD spectroscopy

Far-UV CD spectra of WT and mutant BldC proteins were recorded on an AVIV 435 CD Spectrophotometer in a 1 mm sample cell. Measurements were taken from 200 to 260 nm with a wavelength step of 1.0 nm and a 1 s averaging time. Each spectrum is the average of 5 scans. Protein concentrations ranged from 0.3 to 1.5 mg/ml (the final spectra were normalized for concentration) and the buffer composition was 20 mM NaH_2_PO_4_ (pH 7.5), 300 mM NaF, 5% glycerol, and 0.5 mM Tris(2-carboxyethyl)phosphine (TCEP).

### Data availability

ChIP-chip data supporting the findings of the study have been deposited at the ArrayExpress database under accession number E-GEOD-28908 [https://www.ebi.ac.uk/arrayexpress/experiments/E-GEOD-28908/]. Structure factor amplitudes and coordinates for the BldC-*whiI* opt and BldC-*smeA-ssfA* structures have been deposited in the RCSB Protein Data Bank under the accession codes 6AMK and 6AMA. All other relevant data supporting the findings of the study are available from the corresponding authors upon reasonable request.

## Electronic supplementary material

Supplementary Information(PDF 13493 kb)

Description of Additional Supplementary Files(PDF 245 kb)

Supplementary Data 1(XLSX 50 kb)
