# Supplementary material for: The MerR-like protein BldC binds DNA direct repeats as cooperative multimers to regulate Streptomyces development
Source: Nat Commun. 2018 Mar 19;9:1139. doi: 10.1038/s41467-018-03576-3 (PMC5859096; doi:10.1038/s41467-018-03576-3)
Supplement: Supplementary file 1 — Supplementary Information(PDF 13493 kb) [file 41467_2018_3576_MOESM1_ESM.pdf]

# SUPPLEMENTARY INFORMATION

**Schumacher *et al.* The MerR-like protein BldC binds DNA direct repeats as cooperative multimers to regulate *Streptomyces* development**

## Supplementary Figure 1

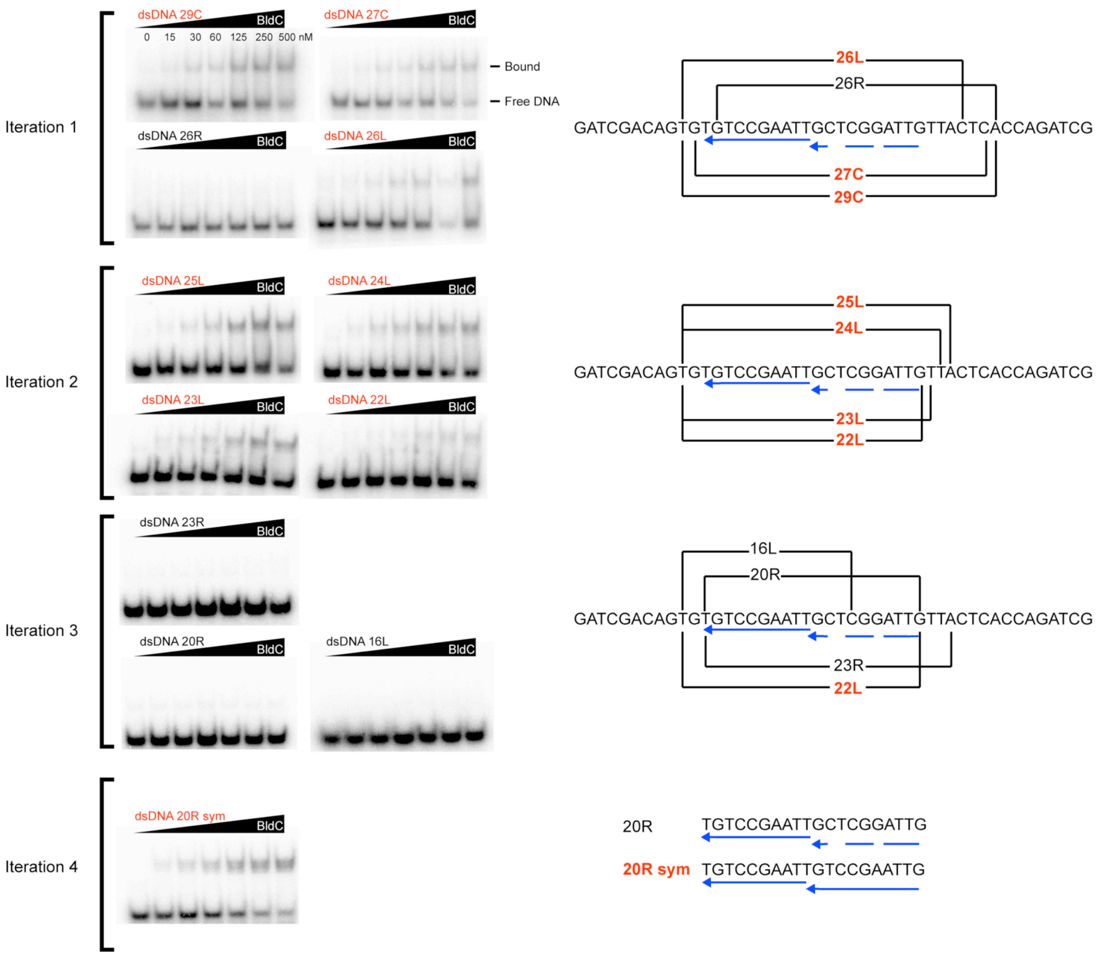


**Supplementary Figure 1 | Identification of the minimal, optimized BldC operator sequence in the *whiI* promoter.** Serially truncated double-stranded oligomers were used in an electrophoretic mobility shift assay to define the minimal *whiI* operator DNA that allows BldC binding. Bands corresponding to the protein-DNA complex (Bound) and free DNA are indicated. The final concentrations of BldC used throughout the figure are indicated above the top left-hand panel. Double-stranded DNAs that allow BldC binding are highlighted in red. We defined DNA 22L that encompasses the imperfect direct repeat as the minimal operator for BldC. Mutagenesis of the 22L sequence to construct a perfect direct repeat allowed the BldC operator to be shortened by two nucleotides (DNA 20R sym) while retaining strong interaction with BldC. The DNA sequence of 20R sym was subsequently used for crystallization of the BldC-*whiI* opt DNA complex and the structure showed that the protein binds as an asymmetric dimer with an N-term to C-term (head to tail) directionality on the DNA according to the directions of the blue arrows.

**Supplementary Figure 2**

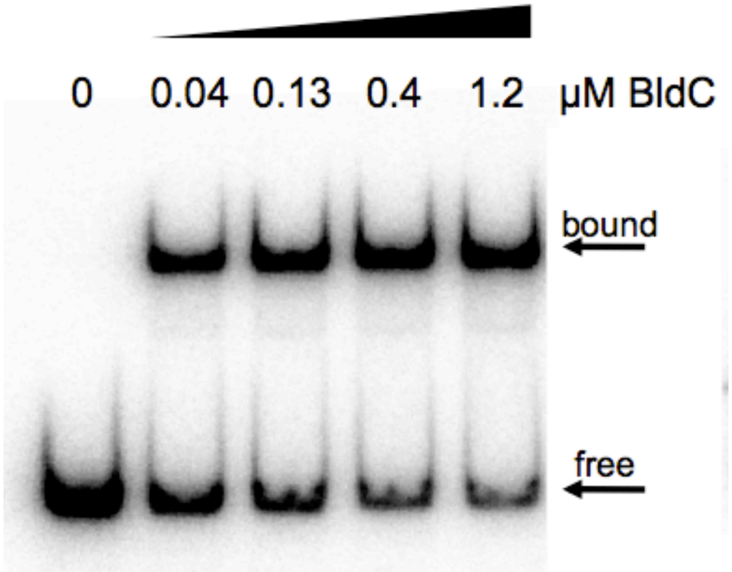


**Supplementary Figure 2 | Electrophoretic mobility shift assay showing BldC binding to its operator in the *smeA* promoter**. Bands corresponding to the protein-DNA complex (bound) and free DNA are indicated. The final concentrations of BldC used are indicated above the lanes.

**Supplementary Figure 3**

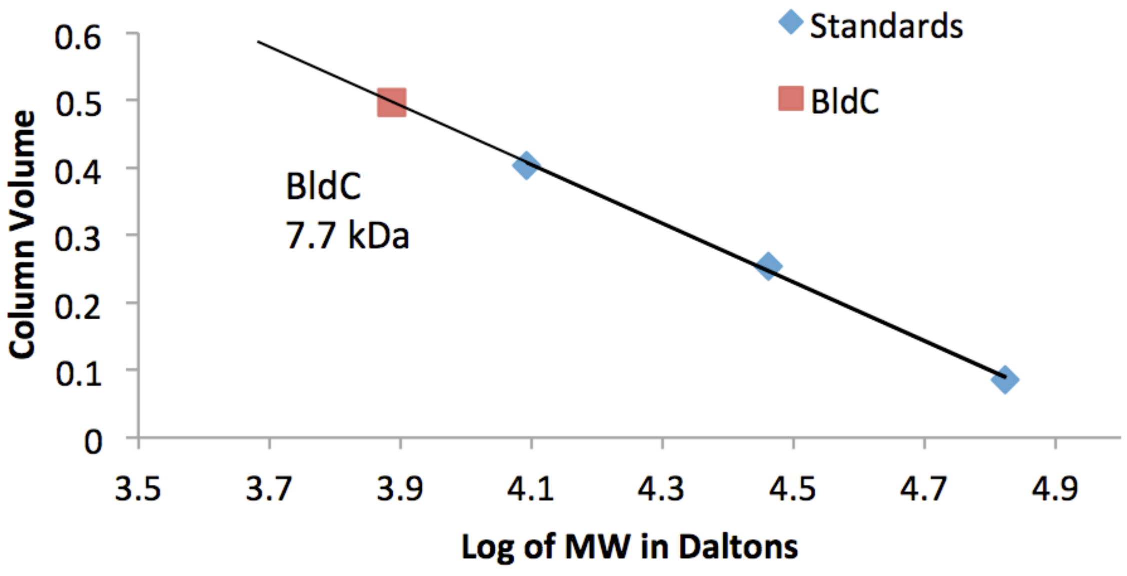


**Supplementary Figure 3 | Size exclusion chromatography (SEC) analysis of apo BldC.** The y-axis is the elution volume normalized for column volume and the x-axis is the log of the MW (in daltons). Shown as blue diamonds are the log MW of the control proteins used to generate the standard curve. These include cytochrome C (12.4 kDa), carbonic anhydrase (29.0 kDa) and serum albumin (66.5 kDa). The calculated MW for BldC from the experiment is 7.7 kDa, which is consistent with the MW of a BldC monomer (7.5 kDa).

**Supplementary Figure 4**

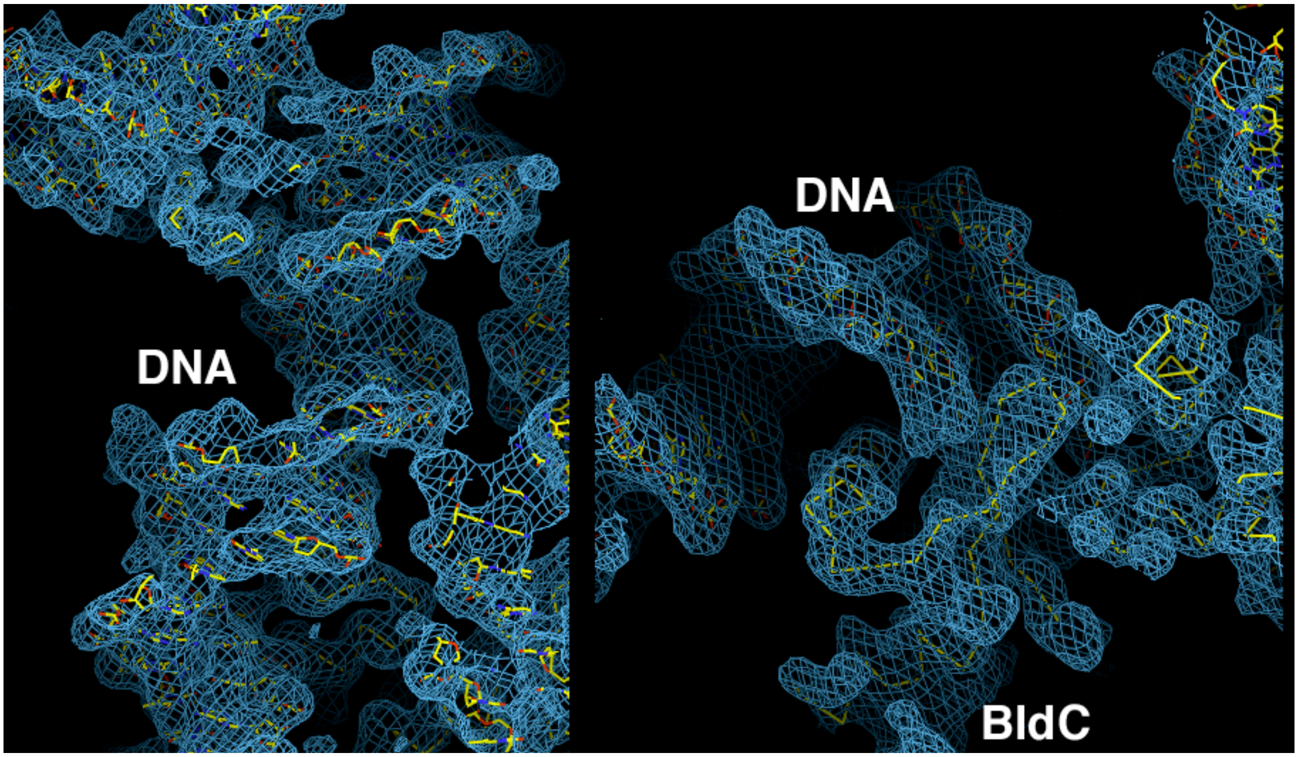


**Supplementary Figure 4 | Experimental SAD electron density map (blue mesh) contoured at 1.3 σ and calculated to 3.28 Å resolution.** Clear electron density is observed for the protein and DNA. The DNA is shown as sticks while the Cα backbone of the protein (right panel) is shown as connected yellow lines.

**Supplementary Figure 5**

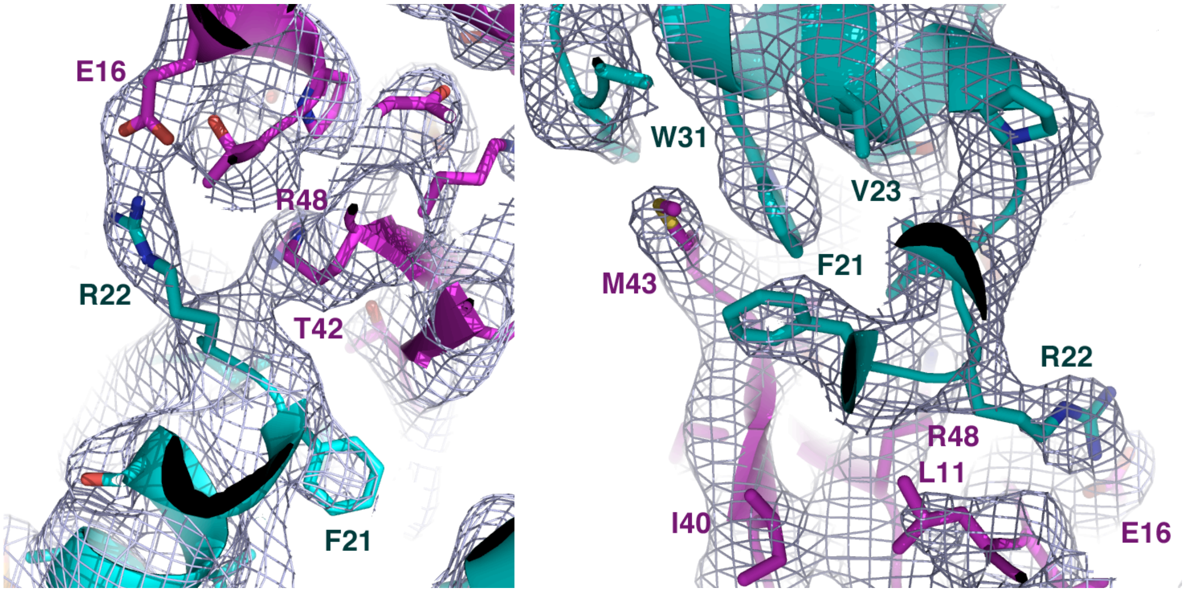


**Supplementary Figure 5 | Representative electron density for the BldC-*whiI* opt structure around the dimerization region.** Views of 2Fo-Fc Sigma-A weighted electron density maps (grey mesh) around the BldC dimer interface. The BldC subunits are coloured cyan and magenta and the maps are contoured at 1.2 σ. Key dimer residues include Arg22 and Glu16, which make a stabilizing salt bridge and hydrophobic residues Leu11, Phe21, Val23, Trp31 and Met43. Residue 43 is a leucine in the WT BldC but was replaced by a selenomethionine in the $P6_122$ crystal form to allow SAD phasing of the BldC-*whiI* opt structure. Note, selenomethionine, methionine and leucine at position 43 support BldC dimerization and DNA binding as revealed in the structures and fluorescence polarization (FP)-based DNA binding analyses (see Fig. 4f).

**Supplementary Figure 6**

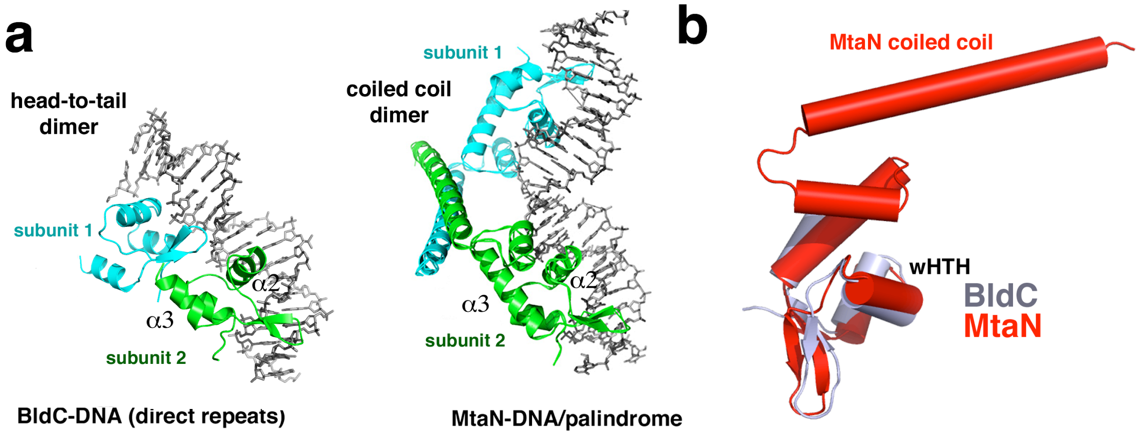


**Supplementary Figure 6 | BldC contains a MerR-like wHTH but displays a different DNA binding mechanism.** (a) Comparison of BldC-DNA (BldC-*whiI*-opt) structure with the structure of the canonical MerR protein MtaN bound to DNA (pdb code: 1R8D). In the structures the green subunit is shown in the same orientation to underscore the difference in DNA binding modes; BldC binds DNA direct repeats as a head-to-tail dimer while MtaN binds palindromic DNA as a symmetric dimer. (b) Superimposition of the BldC DNA binding domain (pink) onto that of the *B. subtilis* MtaN protein (red). The domains can be overlaid with a root mean squared deviation (rmsd) of 1.7 Å for 45 corresponding Cα atoms.

**Supplementary Figure 7**

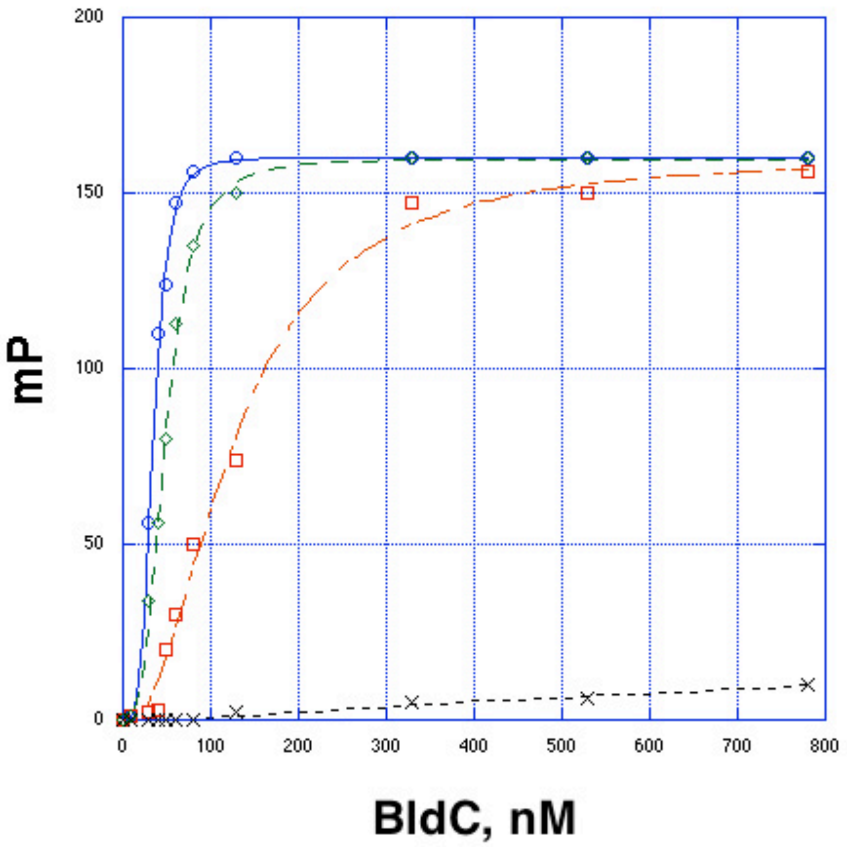


**Supplementary Figure 7 |** FP binding isotherms of BldC binding to WT and mutant fluorescently labeled *whiI* opt 22mer sites. Experiments were preformed as technical triplicates and the error between measurements noted. $His_6$-BldC binding to the WT *whiI* opt site (blue circles), the *whiI* opt site with bps 8 and 17 changed to T (green diamonds) or C (red squares) and to a *whiI* opt site in which both AATT motifs are replaced by GGCC (black crosses). The resulting $K_d$s calculated from these curves are 20.0 ± 8.8 nM, 50.0 ± 1.1 nM, 115 ± 10.0 nM and nonsaturable binding (NB), respectively. The y-axis and x-axis are millipolarization (mP) and BldC concentration, respectively.

**Supplementary Figure 8**

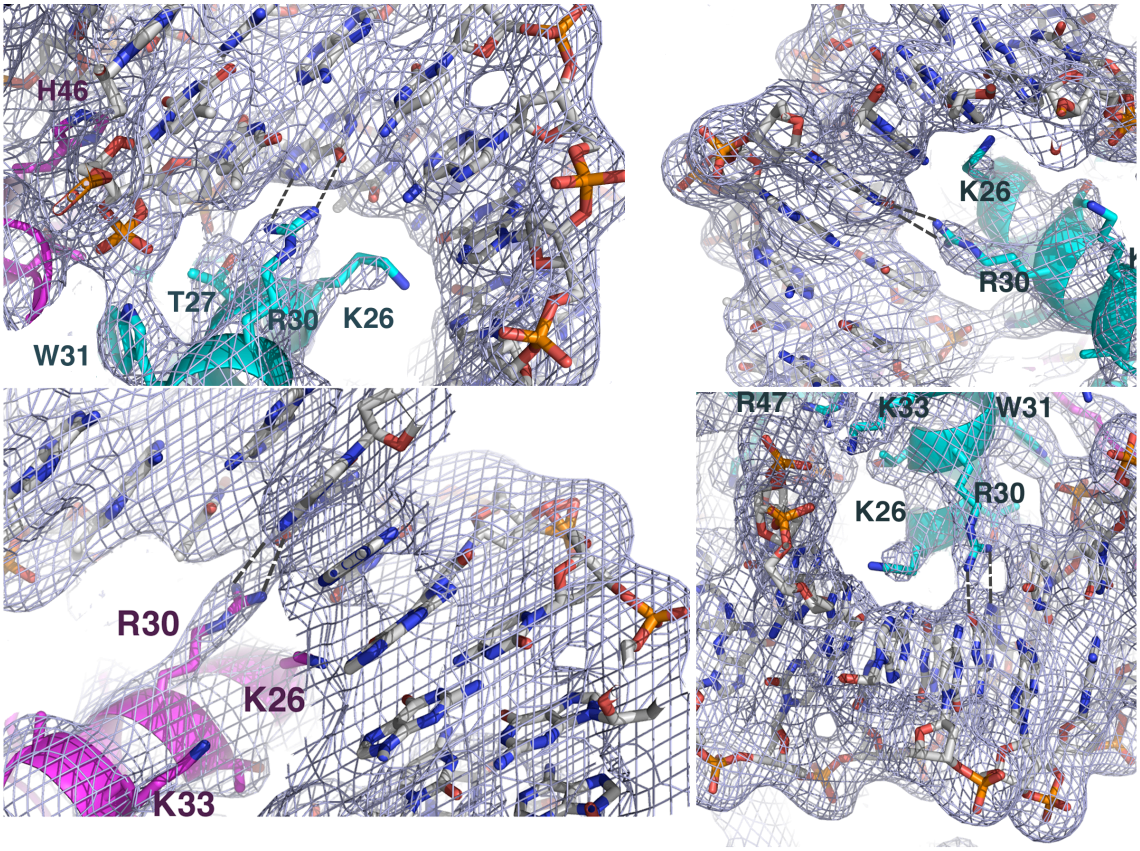


**Supplementary Figure 8 | Representative electron density for the BldC-*whiI* opt structure around DNA binding residues.** Shown is a 2Fo-Fc Sigma-A weighted map (grey mesh) contoured at 1.0 σ to 3.28 Å resolution around key residues that mediate DNA contacts in the structure. The density for residue Arg30 is clear, showing that it specifically reads the guanine base and stacks with the thymine 5´ to the guanine. The two BldC subunits are coloured cyan and magenta.

**Supplementary Figure 9**

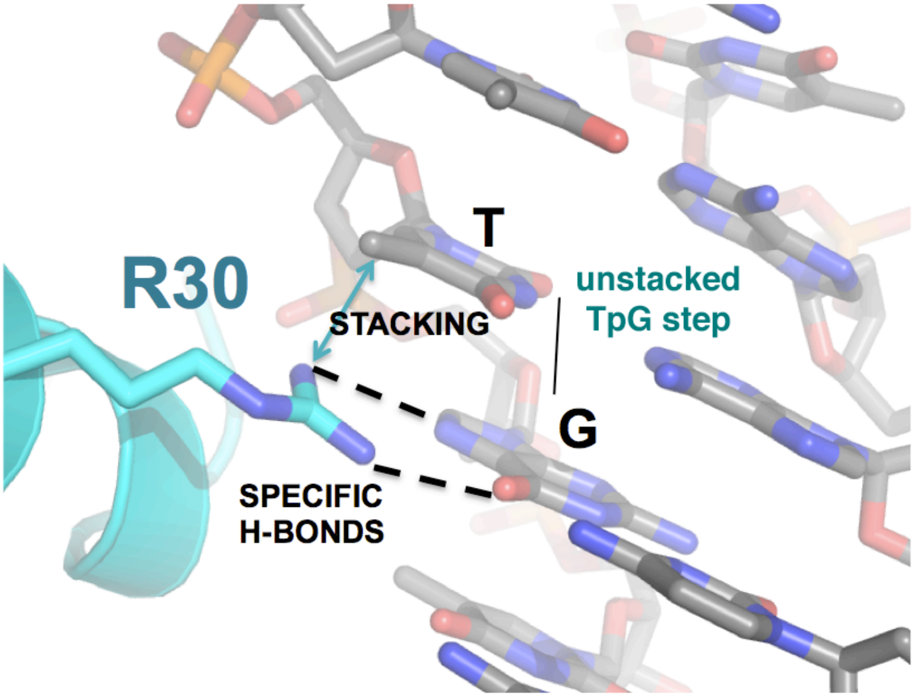


**Supplementary Figure 9 | Close up of the BldC Arg30-YpG interaction.** This special type of base specific contact involves the simultaneous formation of specific hydrogen bonds from an arginine side chain to a guanine base and stacking contacts between the arginine guanidinium group and the preceding 5´ pyrimidine that unstacks from this guanine base.

**Supplementary Figure 10**

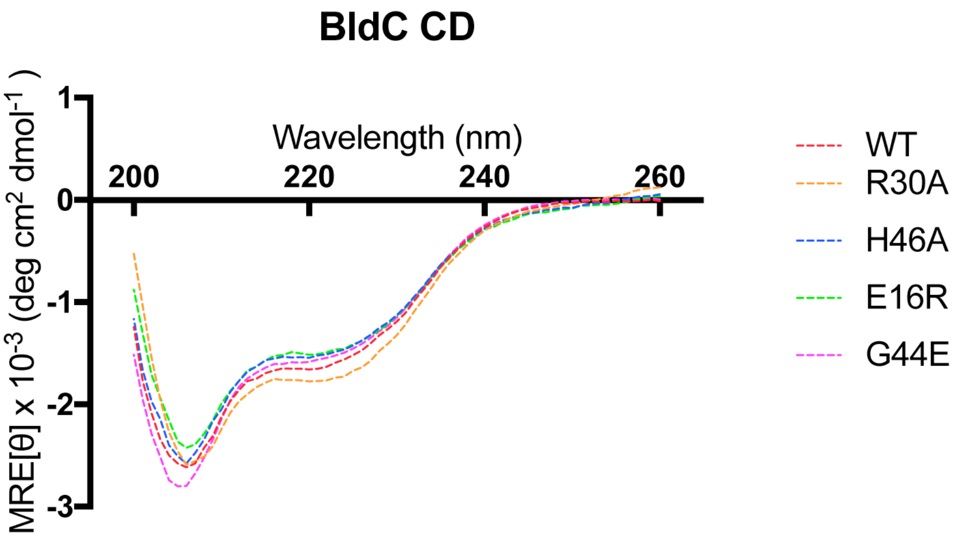


**Supplementary Figure 10 | Circular dichroism (CD) spectra of WT BldC and BldC mutants utilized in FP binding studies.** The spectra overlap, indicating that the mutants are folded and also harbor the same overall fold as the WT protein.

**Supplementary Figure 11**

**a**

```
                             *    *    * *       *           ***
S. coelicolor BldC MTARTPDAEPLLTPAEVATMFRVDPKTVTRWAKAGKLTSIRTLGGHRRYREAEVRALLAGIPQQRSEA
S. avermitilis     MTARTPDAEPLLTPAEVATMFRVDPKTVTRWAKAGKLTSIRTLGGHRRYREAEVRALLAGIPQQRSEA
C. diptheriae        MAREDNGSFLTVAEVAEIMRVSKMTVYRLVHSGELPAVRVGRSFRVHEKAVNEYLDSSYYNVG
B. thuringensis           MSKLLTAQEVADLLRVHKKTIHRMIHSGKLDASKVANKFLIKEEDAKGLLENKNKLDN
P. gingivalis   MMTEKNLKKEEQIYYDAFDVARILRIHFKTALRWGRNGKLPSFKIGNRRYFPAEGILSYKKTIW
S. meliloti             MKITDPLLTVRESAEVLQISVPTFWRHVADGTLPRPVKIGGLSRWPQSEIIAVIERAKASRSNVA
WP_034266952       MTARTPDAEPLLTPAEVATMFRVDPKTVTRWAKAGKLTSIRTLGGHRRYREAEVRALLAGIPAQREG
WP_017621302       MSTRTPEAEPLLTPAEVATMFRVDPKTVTRWAKAGKLTSIRTLGGHRRYRETEVRALLAGIPNQRSE
WP_018349582       MASRTHEPEPLLTPAEVASMFRVDPKTVTRWAKAGKLSAIRTLGGHRRYRESEVRALLQGQIPQQRQGE
OLB79297           MASRTHEPEPLLTPAEVASMFRVDPKTVTRWAKAGKLSAIRTLGGHRRYRESEVRALLQGQIPQQRVGD
WP_089396266       MISSTADTEVLLTPAEVANMFRVDPKTVTRWAKAGKLSSIRTLGGHRRYRESEVRNLLADVESERGAAag
WP_056593121       MNTHPVETEALLTPSEVATLFRVDPKTVTRWAKAGKLSSIRTLGGHRRYRESEVRDLLNGVPQQRPSDg
WP_048344020       MTVKPAPTDNLLTPSEVATLFRVDPKTVTRWAKAGKLSSIRTLGGHRRYLESEVQALLQGIPQQRAVS
WP_046529150       MSTHSSETEALLTPSEVATLFRVDPKTVTRWAKSGKLSSIRTLGGHRRYRESEVRDLLNGVPQPRGTDa
WP_056884367       MVTRTTEVEKLLTPAEVASLFRVDPKTVTRWAKAGKLNSIRTLGGHRRYREAEVRALLSDVI
```

**b**

```
                 *    *    * *       *            ***
BldC   MTARTPDAEPLLTPAEVATMFRVDPKTVTRWAKAGKLT--SIRTLGGHRRYREAEVRALLAGIPQQRSEA
SoxR   MEKKLPRIKALLTPGEVAKRSGVAVSALHFYESKGLIT--SIRNSGNQRRYKRDVLRYVAIIKIAQRIGIPLATI-
ZntR             MYRIGELAKLADVTPDTIRYYEKQQMMDHD-IRTEGGFRLYSDNDLQRLRFIRYARQLGFTLEAI-
CueR              MNISDVAKITGLTSKAIRFYEEKGLVTPP-MRSENGYRTYTQQHLNELTLLRQARQVGFNLEES-
MtaN            MKYQVKQVAEISGVSIRTLHHYDNIELLNPS-ALTDAGYRLYSDADLERLQQILFFKEIGFRLDEI-
MerR       MEKNLENLTIGVFAKAAGVNVETIRFYQRKGLLPEP-DKPYGSIRRYGEADVTRVRFVKSAQRLGFSLDEI-
TipAL           VSYSVGQVAGFAGVTVRTLHHYDDIGLLVPS-ERSHAGHRRYSDADLDRLQQILFYRELGFPLDEV-
BmrR         MKESYYSIGEVSKLANVSIKALRYYDKIDLFKPAYVDPDTSYRYYTDSQLIHLDLIKSLKYIGTPLEEM-
RacA              MNTNMVASELGVSAKTVQRWVKQLNLP--AERNELGHYSFTAEDVKVLKSVKKQISEGTAIQDI-
TnrA MSTNEASYRDKKVMSIGIVKELTGLSERQIRYYEKRSLLF--PDRTNTGIRKYSFSDVERLMDIADRIEEGVQTSEI-
```

**Supplementary Figure 11 | Sequence alignments of BldC with BldC-like and MerR proteins.** (a) Multiple sequence alignment of small, BldC-like proteins. Like *S. coelicolor* and *S. venezuelae* BldC (which are identical) these proteins only consist of a wHTH domain. Residues that are highly conserved are colored yellow. Asterisks denote the positions of the residues involved in head-tail BldC dimer formation. Residues highlighted in green are involved in formation of the hydrophobic core of the head-tail dimer in the *S. coelicolor* BldC. These residues are all hydrophobic in these proteins. The residues involved in the Glu16 to Arg22 cross-dimer contact are highlighted in cyan. The *P. gingivalis* and *S. meliloti* proteins contains an Asp-Arg and Glu-Gln pair, both of which could form cross-dimer contacts. The BldC-like proteins included are; WP_034266952, *Actinospica robiniae*; WP_017621302, *Nocardiopsis gilva*; WP_018349582, *Longispora albida*; OLB79297, *Actinobacteria bacterium* 13_2_20CM_2_71_6; WP_089396266, *Micrococcales bacterium* KH10; WP_056593121, *Cellulomonas* sp. Leaf334; WP_048344020, C. sp. A375-1; WP_041940453, *Frankia alni*; WP_046529150, *Cellulomonas*; WP_056884367, *Phycicoccus* sp. Soil748. (b) Sequence alignments of BldC with classical MerR proteins and the MerR-like *B. subtilis* RacA and TnrA proteins. Classical MerR proteins are characterized by a conserved valine and tyrosine in their HTH motifs and an RXY motif in their wing. Note, these proteins contain hydrophilic or polar residues in the place of the hydrophobic residues that generate the hydrophobic core in the BldC head-tail dimer. They are also missing the cross-contacts (Glu16-Arg22), consistent with the fact that these proteins form symmetric dimers that are very different from the head-to-tail dimer formed by BldC.

## Supplementary Table 1 | List of BldC target genes predicted to encode regulatory proteins

| Serial | LocusTag | Product | Distance | AdjustedPvalue |
|---|---|---|---|---|
| 1 | SCO4768 | BldM | 61 | 0.002132 |
| 2 | SCO0466 | AraC family transcription regulator | 34 | 0.002178 |
| 3 | SCO5556 | HupS | 98 | 0.002528 |
| 4 | SCO5033 | OxyR | 98 | 0.002743 |
| 5 | SCO4410 | anti anti sigma factor | 98 | 0.002817 |
| 6 | SCO3068 | RNA polymerase sigma factor SigI | 124 | 0.003169 |
| 7 | SCO1262 | GntR family transcriptional regulator | 77 | 0.003631 |
| 8 | SCO4035 | RNA polymerase sigma factor SigF | 159 | 0.003852 |
| 9 | SCO0942 | RNA polymerase sigma factor | 82 | 0.00387 |
| 10 | SCO3583 | integral membrane regulator | 34 | 0.00387 |
| 11 | SCO5170 | TetR family transcriptional regulator | 93 | 0.004082 |
| 12 | SCO5811 | transcriptional regulator | 50 | 0.004086 |
| 13 | SCO3034 | WhiB | 43 | 0.005157 |
| 14 | SCO3348 | DNA-binding protein | 137 | 0.006816 |
| 15 | SCO5403 | two-component system response regulator | 78 | 0.00703 |
| 16 | SCO4871 | TetR family transcriptional regulator | 183 | 0.007801 |
| 17 | SCO4232 | transcriptional factor regulator | 146 | 0.01101 |
| 18 | SCO6664 | transcriptional regulator | 82 | 0.0167 |
| 19 | SCO4091 | BldC | 114 | 0.01893 |
| 20 | SCO0132 | transcriptional regulator | 98 | 0.02572 |
| 21 | SCO4754 | transcriptional regulator | 34 | 0.02601 |
| 22 | SCO4897 | transcriptional regulator | 34 | 0.03031 |
| 23 | SCO2075 | DNA-binding protein | 40 | 0.03159 |
| 24 | SCO3810 | GntR family transcriptional regulator | 156 | 0.032 |
| 25 | SCO1568 | TetR family transcriptional regulator PqrA | 38 | 0.04402 |

**For each target, their gene identifiers (LocusTag), predicted function (Product) based on annotation in strepdb (http://strepdb.streptomyces.org.uk), distance to an enriched probe (Distance) and the corresponding P-value (AdjustedPValue) of the enriched probe is listed.**

## Supplementary Table 2 | List of BldC targets that are also BldD targets

| Serial | LocusTag | Product |
|---|---|---|
| 1 | SCO1414 | SffA |
| | SCO1415 | SmeA |
| 2 | SCO2062 | hypothetical protein |
| | SCO2063 | putative small hydrophilic protein |
| 3 | SCO2528 | 2-ispoprylmalate synthase LeuA |
| | SCO2529 | putative metalloprotease. |
| 4 | SCO2817 | conserved hypothetical protein |
| | SCO2818 | conserved hypothetical protein |
| 5 | SCO3034 | WhiB |
| 6 | SCO3862 | putative membrane protein |
| | SCO3863 | putative secreted protein |
| 7 | SCO4091 | BldC |
| 8 | SCO4175 | hypothetical protein |
| | SCO4176 | conserved hypothetical protein |
| 9 | SCO4239 | putative small membrane protein |
| 10 | SCO4767 | WhiD |
| | SCO4768 | BldM |
| 11 | SCO4796 | hypothetical protein |
| | SCO4797 | putative ATP-dependent DNA helicase II |
| 12 | SCO5366 | ATP synthase protein I |
| 13 | SCO5544 | CvnA1 |
| | SCO5545 | hypothetical protein |
| 14 | SCO5810 | transmembrane efflux protein |
| 15 | SCO5970 | hypothetical protein |

**For each target, their gene identifiers (LocusTag) and predicted function (Product) based on annotation in strepdb (http://strepdb.streptomyces.org.uk) is listed.**

**Supplementary Table 3 | Data collection and refinement statistics for the BldC-DNA structures**

| Structure | BldC-*whiI* opt | BldC-*smeA-ssfA* |
|---|---|---|
| Pdb Code | 6AMK | 6AMA |
| Space Group | $P6_122$ | $P4_122$ |
| Cell dimensions | | |
| a,b,c (Å) | 114.2, 114.2, 120.0 | 159.3, 159.3, 130.8 |
| α,β,γ (°) | 90, 90, 120 | 90, 90, 90 |
| Resolution (Å) | 98.7-3.28 | 130.8-3.09 |
| Total reflections, # | 88362 | 191414 |
| Unique reflections, # | 9331 | 31106 |
| $R_{sym}$ | 0.116 (1.438)* | 0.118 (2.115) |
| $R_{pim}$ | 0.055 (0.670) | 0.051 (0.905) |
| CC(1/2) | 0.990 (0.665) | 0.997 (0.354) |
| I/σI | 11.7 (2.1) | 8.0 (1.0) |
| Completeness (%) | 99.4 (99.2) | 99.2 (100.0) |
| Redundancy | 9.5 (9.8) | 6.2 (6.3) |
| Refinement | | |
| Resolution (Å) | 98.7-3.28 | 130.8-3.09 |
| $R_{work}/R_{free}$ (%) | 22.3/26.8 | 20.9/27.8 |
| RMS deviations | | |
| Bond lengths (Å) | 0.006 | 0.003 |
| Bond angles (°) | 1.231 | 0.838 |
| Ramachandran analyses | | |
| Favored (%) | 95.0 | 92.0 |
| Disallowed (%) | 0.0 | 0.0 |

***Values in parentheses are for highest-resolution shell.**

## Supplementary Table 4 | Strains, plasmids and oligonucleotides used in this study

| | Relevant genotype/comments | Source or reference |
|---|---|---|
| Strains | | |
| *S. coelicolor* | | |
| M600 | Prototroph, SCP1$^-$, SCP2$^-$ | 42 |
| J2166 | *ΔbldC* derivative of M600 | 16 |
| | | |
| *E. coli* | | |
| BL21(DE3) | Strain used to overexpress his-BldC | |
| | | |
| Plasmids | | |
| pIJ6838 | pET15b expressing full-length his-tagged BldC | 16 |
| | | |
| Oligonucleotide | | |
| 6029_F1 | GAGAACGGACACCAGGCCTC | |
| 6029_R1 | CCAGCCGTCGTTCATCGTTC | |
| 1415_F2 | GTGAACCGCCAGGGTACTGG | |
| 1415_R2 | CGAGAGGATCATGTGAGTTC | |
