# Supplementary material for: The MerR-like protein BldC binds DNA direct repeats as cooperative multimers to regulate Streptomyces development
Source: Nat Commun. 2018 Mar 19;9:1139. doi: 10.1038/s41467-018-03576-3 (PMC5859096; doi:10.1038/s41467-018-03576-3)
Supplement: Supplementary file 2 — Description of Additional Supplementary Files(PDF 245 kb) [file 41467_2018_3576_MOESM2_ESM.pdf]

Description of Additional Supplementary Files

File Name: Supplementary Data 1

Description: ChIP-chip data set for *S.coelicolor* BldC. Each row represents an enriched probe (probeID) with the mid-position (midpos) of each probe on the *S. coilicolor* genome recorded. Enrichment ratios for the wild type, relative to the *bldC* mutant are expressed as the log-fold change (logFC). Probes are listed in order of significance (adjusted p value - adj.P.Val). For each probe, the nearest gene to the left and right is identified (left/rightLocusTag), its distance to the midpos of the probe (left/rightDistance), whether the gene is on the forward (1) or reverse (-1) strand (inStrand) and the predicted function (left/rightProduct) based on annotation in strepdb (http://strepdb.streptomyces.org.uk). If the midpos of a probe falls within a gene, it's gene identifier (inLocusTag), distance to the probe (inDistance), whether the gene is on the forward (1) or reverse (-1) strand (inStrand) and predicted function (inProduct) is also listed.
